# MIDA: A Multimodal Imaging-Based Detailed Anatomical Model of the Human Head and Neck

**DOI:** 10.1371/journal.pone.0124126

**Published:** 2015-04-22

**Authors:** Maria Ida Iacono, Esra Neufeld, Esther Akinnagbe, Kelsey Bower, Johanna Wolf, Ioannis Vogiatzis Oikonomidis, Deepika Sharma, Bryn Lloyd, Bertram J. Wilm, Michael Wyss, Klaas P. Pruessmann, Andras Jakab, Nikos Makris, Ethan D. Cohen, Niels Kuster, Wolfgang Kainz, Leonardo M. Angelone

**Affiliations:** 1 Division of Biomedical Physics, Office of Science and Engineering Laboratories, Center for Devices and Radiological Health, US Food and Drug Administration, Silver Spring, Maryland, 20993, United States of America; 2 IT'IS Foundation for Research on Information Technologies in Society, Zurich, Switzerland; 3 Swiss Federal Institute of Technology (ETH) Zurich, 8092 Zurich, Switzerland; 4 Institute for Biomedical Engineering, University of Zurich and ETH Zurich, Zurich, Switzerland; 5 Computational Imaging Research Laboratory, Department of Biomedical Imaging and Image-guided Therapy, Medical University of Vienna, Austria; 6 Computer Vision Laboratory, ETH Zurich, Zurich, Switzerland; 7 Athinoula A. Martinos Center For Biomedical Imaging, Department of Radiology, Massachusetts General Hospital, Harvard Medical School, Charlestown, Massachusetts, 02129, United States of America; 8 Center for Morphometric Analysis, Department of Psychiatry and Neurology, Massachusetts General Hospital, Harvard Medical School, Charlestown, Massachusetts, 02129, United States of America; Aarhus University, DENMARK

## Abstract

Computational modeling and simulations are increasingly being used to complement experimental testing for analysis of safety and efficacy of medical devices. Multiple voxel- and surface-based whole- and partial-body models have been proposed in the literature, typically with spatial resolution in the range of 1–2 mm and with 10–50 different tissue types resolved. We have developed a multimodal imaging-based detailed anatomical model of the human head and neck, named “MIDA”. The model was obtained by integrating three different magnetic resonance imaging (MRI) modalities, the parameters of which were tailored to enhance the signals of specific tissues: i) structural T1- and T2-weighted MRIs; a specific heavily T2-weighted MRI slab with high nerve contrast optimized to enhance the structures of the ear and eye; ii) magnetic resonance angiography (MRA) data to image the vasculature, and iii) diffusion tensor imaging (DTI) to obtain information on anisotropy and fiber orientation. The unique multimodal high-resolution approach allowed resolving 153 structures, including several distinct muscles, bones and skull layers, arteries and veins, nerves, as well as salivary glands. The model offers also a detailed characterization of eyes, ears, and deep brain structures. A special automatic atlas-based segmentation procedure was adopted to include a detailed map of the nuclei of the thalamus and midbrain into the head model. The suitability of the model to simulations involving different numerical methods, discretization approaches, as well as DTI-based tensorial electrical conductivity, was examined in a case-study, in which the electric field was generated by transcranial alternating current stimulation. The voxel- and the surface-based versions of the models are freely available to the scientific community.

## Introduction

Computational modeling is being increasingly used by industry, government, and academia to complement experimental testing for safety and efficacy of medical devices. Modeling contributes to the creation of personalized medicine, as it facilitates disease diagnosis, planning of pharmaceutical and surgical interventions, predicting treatment outcomes, and optimizing clinical treatment [1]. Furthermore, computational modeling and simulations offer access to full three-dimensional (3D) data and quantities that can be hard to access by experimental measurement. Complex models of human anatomy have been used for dosimetric purposes to compute the tissue absorption from external ionizing radiation sources [2–6] and internally deposited radioactive sources [7–10] by tissues. Anatomical models have also been used to calculate the energy absorption and temperature increase in tissues exposed to electromagnetic fields [11–13], to calculate the current densities generated by low-frequency exposure [14–18], to assess the biomechanical behavior of the musculoskeletal system [19], as well as in design and investigation of the underlying mechanisms of transcranial focused ultrasound [20,21].

There are over twenty whole and partial body image-based models of adults reported in the literature [4,6,7,11,15,16,22–43]. Reconstruction of cryosection images from cadavers has been proposed as an approach to catalogue gross human anatomy and generate computational human models [26,29,30,37,38,44–46]. The Visible Photographic Man (VIP-Man) model [29] of the Visible Human Project was generated by segmenting cryosections of an *ex vivo* human body and was originally proposed for Monte Carlo radiation dose calculation. This model included over 31 head structures. The Visible Chinese Human *ex vivo* data were used to generate two models, including 37 [47] and 49 head structures [48]. Cryosection data offer better visualization of the tissues than *in vivo* images, however, *ex vivo* preparations can induce changes in the configuration of anatomical samples, including deformation of the tissues, collapse of the vessels due to the loss of blood pressure, and uneven distributions of the intracranial fluids [45,49]. A further drawback of the *ex vivo* technique is that the digital photographs taken from each tissue section are limited to two dimensions, introducing uncertainties in the 3D spatial reconstruction of the structure.

With the advancement of high resolution 3D medical imaging modalities, magnetic resonance imaging (MRI) and computed tomography (CT) have become other fundamental sources of information for reconstruction of the human anatomy. The MRI and CT data of the Visible Human Project were used to generate the HUGO model [30]. This model has 1 mm isotropic resolution, includes 15 different structures in the head, and was originally proposed for electromagnetic and thermal analyses. Makris et al. [41] presented an MRI-based model of the head at 1 mm isotropic spatial resolution. The Virtual Family [40] was originally generated from whole-body MRI images of four models containing up to 39 different structures in the head [40] and has been more recently extended to ten models, including elderly male and obese male models, for expanded population coverage [50]. Finally, Segars et al. [42] have introduced a hybrid model of the head with about 50 different structures. This model was based on a 1 mm isotropic resolution MRI dataset segmented to generate a voxelized model with non-uniform rational B-splines (NURBS) to define each anatomical object.

In our study, we have developed a multimodal imaging-based detailed anatomical (MIDA) model of the head and neck, segmented at 500 μm isotropic resolution, which includes 153 structures. The model includes the registration and integration of data from three MRI modalities: a) data on structural images: T1- and T2-weighted MRIs were acquired to image the brain, bone and soft-tissues over the entire head; additionally, a heavily T2-weighted MRI sequence with high nerve contrast was optimized to enhance the slab containing the ear and eye regions with their associated cranial nerves; b) data on vasculature: time-of-flight (TOF) and phase-contrast (PCA) magnetic resonance angiography (MRA) were performed to image and distinguish arteries and veins; and c) data on tissue anisotropy and fiber orientation: diffusion tensor imaging (DTI) of the water in the brain was performed to provide information about the fibrous nature of brain tissues.

## Materials and Methods

### Data Acquisition

Scans of the head and neck down to the level of the fifth cervical vertebra (C5) of one healthy 29-year old female volunteer were acquired at the Institute for Biomedical Engineering (ETH, Zurich, Switzerland). All the images were acquired on a PHILIPS Achieva 3 Tesla MRI scanner (Philips Healthcare, Best, the Netherlands) with an 8-channel receive-only head coil array. The scanning time for the entire protocol was three hours. Informed consent was obtained in accordance with policies of the Institute for Biomedical Engineering at the ETH.

**Fig 1:**
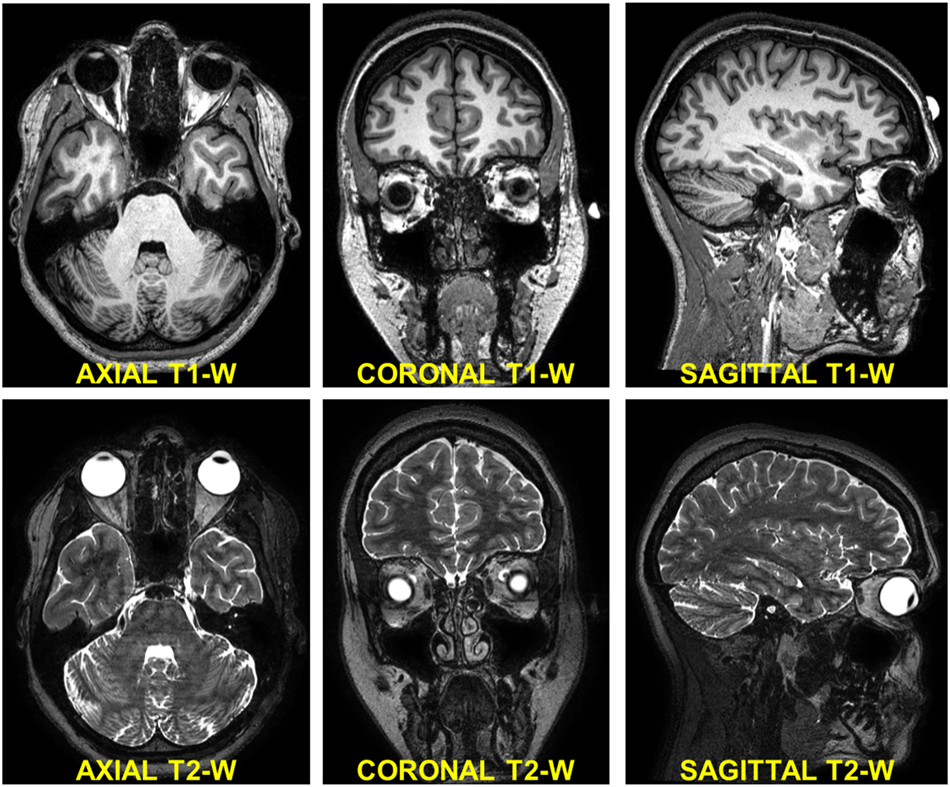
Structural MRI scans used for segmentation. Axial (left), coronal (middle), and sagittal (right) views of the T1- (top) and T2-weighted (bottom) structural MRIs. A specific T2-weighted MRI sequence with high nerve contrast optimized to enhance the structures of the ear and eye was also acquired (data not shown).

Several sequences were performed to obtain high resolution anatomical images with T1 and T2 contrasts: the top half of Fig 1 shows the axial, coronal, and sagittal views of MRI head images from a T1-weighted 3D magnetization prepared gradient echo sequence (MPRAGE) at 500 μm isotropic resolution, TR / TE / flip angle: 15.18 ms / 6.96 ms / 8°, field of view (FOV): 240 mm × 240 mm, number of signal averages (NSA): 1. The bottom half of Fig 1 shows the same views from a T2-weighted 3D turbo spin echo (TSE) sequence with low refocusing flip angle sweep [51] at 500 μm isotropic resolution, TR / TE / TE equivalent to standard TSE: 2250 ms / 424 ms / 164 ms, FOV: 240 mm × 240 mm, NSA: 1. In addition, a heavily T2-weighted standard 3D TSE imaging slab at 500 μm isotropic resolution, TR / TE / flip angle: 1500 ms / 197 ms / 90°, FOV: 180 mm × 180 mm, NSA: 1, SENSE undersampling factor: 2—was acquired to image the eye and the ear regions with improved T2 contrast.

**Fig 2:**
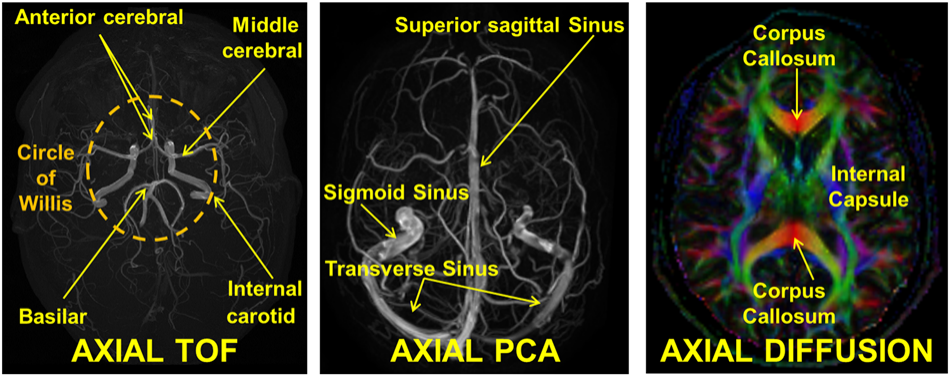
Vasculature information and DTI. Axial view of maximum intensity projection from the 3D TOF (left) and the 3D PCA (middle) MRA. The TOF was optimized to highlight blood flowing in the cranial direction, i.e., mostly arteries, whereas the velocity window of the PCA was chosen such to highlight mostly veins. On the right an axial view of the principal eigenvector map is shown. In the color-coded fiber map, red, green, and blue represent the principal diffusion directions. It is possible to distinguish the corpus callosum in red with its fibers running mostly in left-right direction and the internal capsule bundle in blue with fibers running mostly in superior-inferior direction. Diffusion imaging is not directly used for the segmentation and generation of the anatomical model, but it provides anisotropic electrical properties of the tissues for electromagnetic applications and nerve orientation.

To image the vasculature of the head, MRA scans were performed. To distinguish arteries from veins, a 3D TOF sequence at 0.39 mm × 0.39 mm × 0.5 mm resolution, TR / TE / flip angle: 25 ms / 3.45 ms / 20°, FOV: 200 mm × 200 mm, NSA: 1, SENSE undersampling factor: 2 and a 3D PCA sequence at 0.72 mm × 0.72 mm × 0.8 mm resolution, TR / TE / flip angle: 19.09 ms / 7.06 ms / 10°, FOV: 230 mm × 230 mm, NSA: 1, SENSE undersampling factor: 2 were employed. Fig 2 shows axial maximum intensity projections from the 3D TOF (left) and the 3D PCA (middle) volumes. To assess the microstructure of the brain, a diffusion-weighted single-shot spin-echo echo-planar-imaging (DW SSh-SE-EPI) sequence was acquired, with 32 diffusion orientations (*b*-values 0 and 800 s/mm^2^), at 1.5 mm × 1.5 mm × 2.5 mm resolution:, TR / TE: 7121 ms / 85 ms, FOV: 240 mm × 240 mm, NSA: 5, SENSE under sampling factor: 3, spectral fat saturation pre-pulse. An axial view of the principal eigenvector map is given in Fig 2 (right). In the color-coded map, red, green, and blue represent the principal diffusion directions.

### Data Co-Registration

**Fig 3:**
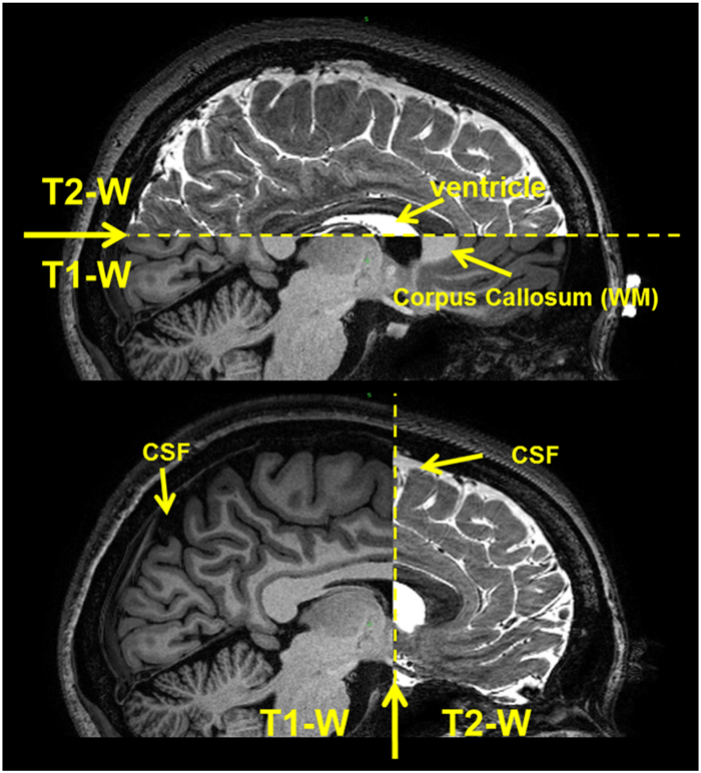
T1- and T2-weighted MRI registration. Sagittal view of the registered and integrated T1- and T2-weighted MRIs. The contrast between WM, e.g., corpus callosum, and GM signals is higher in T1, while the CSF, e.g., ventricle, is enhanced in T2. The tissue segmentation was performed by reaping the benefits of both MRI datasets.

The exact spatial relationship between different datasets acquired on the same subject at different time points may be impaired by inter-scan patient motion, which causes translational and rotational differences in position and orientation between the sets of scans—even when recorded within the same session—and scaling and shearing geometric distortions intrinsic to the scanner. To correct for such differences, each MRI and MRA dataset was resampled to an isotropic resolution of 500 μm, and an intensity-based affine registration [52] with 12 degrees of freedom—three translation, three rotation, three scaling, and three shearing parameters—was used to align them to the reference T1-weighted MRI. The normalized mutual information [53,54] was chosen as the voxel similarity measurement for the registration because it makes no assumptions on the relationship between image intensities, and, therefore, it is the criterion of choice for images that show intensity differences due to different acquisition protocols. Registration allowed the multi-modality head MRI scan types acquired to be integrated into a single representation with one coordinate system, while taking advantage of the redundant and complementary information provided by the different image sources for improvement of the segmentation. A specific non-rigid registration approach was used for the registration of the DTI, as explained in section 6 of the Materials and Methods. Fig 3 shows a sagittal view of the registered and integrated T1- and T2-weighted MRIs.

### Segmentation

The segmentation was performed by three trained experts using the iSeg software (Zurich MedTech, Zurich Switzerland) [55] and switching among the available datasets depending on which showed the best contrast for the specific structure to be outlined. The T1-weighted MRIs were the preferred dataset for segmentation, unless otherwise specified. For each structure the general segmentation procedure was performed on multiple slices at a time, starting with the first and last slices and then segmenting the slices in between using a topologically flexible interpolation. The number of slices used for the interpolation ranged from a minimum of three to a maximum of ten, depending on the geometric complexity of the structure. In general, the following segmentation steps were performed:

1. Image pre-processing for noise filtering using 3D Gaussian smoothing with 1 mm × 1 mm × 1 mm kernel.
2. A preliminary rough segmentation of the first and last slices of the group with automatic algorithms, mostly by means of histogram analysis-based thresholding, region growing [56], interactive watershed transformation and *k*-means clustering [57]. An overview of the semi-automatic segmentation methods provided by the iSeg software and used for the model generation is given in Appendix S1, section 1.
3. A first refinement of these same slices with semi-automatic tools, such as the holes/gap filling tool, island removing tool, and morphological operations such as opening and closing [58].
4. Fine manual adjustments and smoothing of these same slices with a brush of adjustable radius.
5. A topologically flexible interpolation between the first and last slices to automatically segment the tissues in the intervening slices [59] and a final manual refinement.
6. Steps iii. and iv. were repeated, alternating between axial, coronal, and sagittal views.

**Fig 4:**
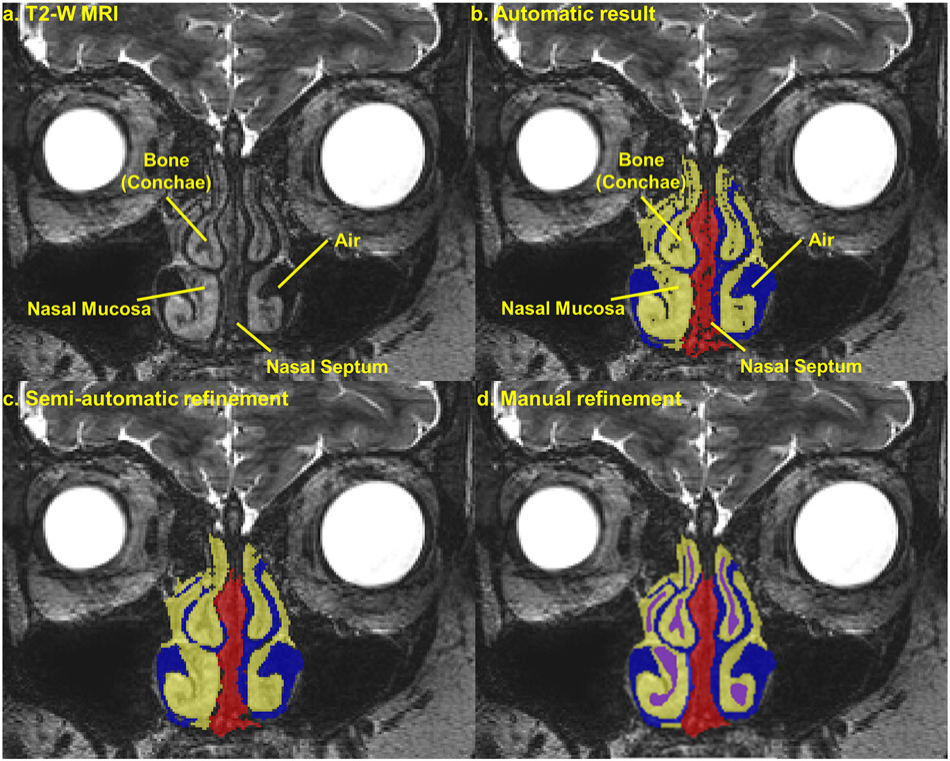
Segmentation procedure. (a) Coronal section of the T2-weighted MRI centered on the nasal region, (b) the result of the automatic segmentation of the nasal mucosa, nasal septum, and air cavity by means of a region growing technique (step ii), (c) the result of the segmentation after the application of semi-automatic smoothing algorithms (step iii), and (d) the final segmentation result after manual delineation of the bone (not captured automatically) and global manual refinement (step iv).

Fig 4 shows the original T2-weighted MRI data and an example of steps ii (b), iii (c), and iv (d) of the segmentation procedure for outlining the nasal mucosa, nasal septum, and the nasal air cavity. A detailed description of the segmentation of the different structures included in the model is provided in S1 Appendix.

To assess inter-operator variability, the segmentations of 35 different structures of the head performed by the three experts (operators A, B, and C) on two axial, two coronal, and two sagittal slices were compared with a reference consensus segmentation (ground truth or GT) obtained with the simultaneous truth and performance level estimation (STAPLE) algorithm [60]. The six slices were randomly picked from the original dataset, as were the 35 head structures contained in the slices. For simplicity, we asked the operators to segment the skull, muscles, and vertebrae as unique structures without discerning between substructures and sub-layers. For each structure, the STAPLE algorithm was used to estimate the unknown ground truth from the outlines of the three operators while providing a measure of the performance level achieved by each operator. Such ground truth represents a hidden consensus segmentation obtained iteratively with estimation and maximization steps by combination of the three operators’ segmentations, each weighted with its associated estimated performance level. The algorithm incorporates also a prior model accounting for homogeneity and spatial constraints of the structures.

The intra-operator variability was assessed similarly. We asked each operator to repeat the segmentations of three selected structures along the entire head volume, i.e., the globus pallidus, putamen, and thalamus, three times. Then, for each operator and each structure, we measured the similarity between the three segmentations performed by the operator on different days and the consensus combination obtained via STAPLE. Globus pallidus, putamen, and thalamus were chosen for repeated segmentation because these structures are relatively small compared with the entire head.

The Dice similarity index [61] and the modified Haussdorf distance [62] were used to quantify inter- and intra-operator variability. The Dice index *D* between segmentation 1 (*S*_*1*_) and segmentation 2 (*S*_*2*_), defined as:

$$D=2\frac{S_{1}\cap S_{2}}{\left|S_{1}\right|+\left|S_{2}\right|}$$ (1)

measures the extent of spatial overlap between *S*_*1*_ and *S*_*2*_. The Dice index ranges between 0 and 1, with 1 signifying perfect agreement between the segmentations. The modified Hausdorff distance *MHD*, which measures the similarity between two shapes, is defined as:

$$MHD\left(S_{1},S_{2}\right)\;=\;\text{max}\left(d\left(S_{1},S_{2}\right),d\left(S_{2},S_{1}\right)\right)$$

$$d(X,Y)=\frac{1}{N_{X}}\sum_{x\in X}\underset{y\in Y}{\min}\left\|x-y\right\|$$ (2)

where || · || denotes the L2-norm and *N*_*X*_ denotes the number of elements in set *X*. Distance values close to 0 correspond to high matching between the boundaries. Analysis of the variance of both the Dice and *MHD* values was performed with the non-parametric Kruskal-Wallis test to assess inter- and intra-operator variability. To complement the quantitative analysis of the segmentation, all 153 segmented structures of the head and neck were extensively reviewed by an expert anatomist.

### Morel Atlas Integration

A special automatic atlas-based process was used for the segmentation of the thalamic nuclei and the red nucleus in the midbrain. Ground truth data for the anatomy of these nuclei were acquired from the 3D adaptation of a multiarchitectonic stereotactical thalamus atlas by Morel [63]. We relied on the extension of the classical two-dimensional atlas with multi-subject data: multiple histological delineations were fused into a statistical shape-model-based digital atlas [64]. The histological procedure of the original atlas generation and anatomical nomenclature is detailed in [63] and [64]. In such shape models, the mean geometry and variability of thalamic nuclei are represented and can be utilized to make predictions about individual subject-specific anatomical configuration, which remains feasible when only partial observations are possible [65]. The procedure described in [66] was followed, and the MRI-visible borders of the thalamus were used to estimate a subject specific map of individual thalamus nuclei. For this step, manual segmentation of the thalamus borders was performed on high-resolution T1-weighted anatomical MRI images. The resulting triangulated meshes of nuclei were projected onto the image grid of the original MRI acquisitions, and volumetric representations were saved.

### Surface Extraction

The voxelized models were transformed to surface descriptions by extraction, smoothing, and simplification of triangulated surfaces, to achieve model sizes that can be reasonably handled. The methodology is based on specially designed algorithms, which allow high-quality triangle elements and topologically conformal surfaces to be ascertained. In the first step, a Delaunay refinement approach is used to generate a tetrahedral mesh from the segmentation [67,68]. A non-uniform sizing field is used to generate smaller triangles in complex thin and narrow tissue. A surface is extracted and further processed, including curvature smoothing [69] and simplification. The simplification collapses short edges and performs edge flips [70] while checking that no self-intersections are introduced.

### Diffusion Tensor Imaging

The recorded DTI images were used to acquire information about the local anisotropic nature of the central nervous system white matter (WM) tissues. The following steps were performed to register the DTI images with the anatomical head model and to compute the fractional anisotropy along with the diffusion tensors for each voxel:

1. Image registration: after initial manual pre-alignment, 3DSlicer [71] was used to register the baseline (diffusion-weighting *b*-value = 0) DTI volume to the T2-weighted image data by means of a non-rigid B-splines registration algorithm based on mutual information to account for distortions from the inhomogeneous static magnetic field. The same algorithm was used to register each different direction dataset on the registered baseline volume to compensate for warping induced by the eddy currents and geometric distortions from the short echo planar-based diffusion-weighted images [72].
2. Reconstruction of the DTI tensor: a MATLAB (MathWorks, Inc., MA, USA) code [73] was used to calculate the diffusion tensors for each voxel.
3. Masking: the DTI tensor data was masked based on the segmented tissue outline to restrict the data to brain tissue voxels.
4. Through dedicated Python routines based on the Visualization ToolKit [74] (VTK, Kitware Inc., New York) for shape function-based interpolation to the simulation mesh and a linear relationship from [75], it was possible to map the DTI tensor data to assign spatially varying tensorial properties to the discretized models to account for location specific tissue anisotropy for simulation purposes.

### Transcranial Alternating Current Stimulation (tACS)

To illustrate the application of the anatomical head model, we performed a simple study of tACS, a technology based on weak sinusoidal currents applied between two electrodes attached to the scalp which potentially allows for noninvasive controlled interference with brain rhythms [76]. Laakso and Hirata have used computational models to investigate possible unwanted tACS-induced visual sensations, known as phosphenes [76]. Based on their work, two electrode configurations, namely two large electrodes placed in the frontalis-vertex (Fpz-Cz) and five small electrodes in a Cz-(Fz, C3, C4, Pz) montage, were modeled. The first configuration was associated with a high phosphene incidence frequency, while the latter configuration led to less visual stimulation and fewer sensations [76]. A rectilinear, structured mesh-based (voxels) quasi-electrostatic solver [14] was used to perform simulations to compare the two setups. In addition, simulations of the Fpz-Cz setup were performed with a novel tetrahedral element-based unstructured finite element modeling (FEM) solver that supports anisotropic electrical conductivity tensors. The goal of this test was to show the applicability of the MIDA head model to different discretization and numerical methods, as well as to illustrate the use of DTI data.

The dielectric material properties were extracted for a stimulation frequency of 10 Hz based on the literature [76–78]. In the unstructured mesh simulations, the dielectric properties inside brain tissues and cerebrospinal fluid (CSF) were correlated by a linear relationship to the DTI tensors according to [79], but with the values capped at 1.8 S/m (CSF conductivity [76]). The rectilinear grids had 65–70 million voxels with resolution as low as 0.14 mm, and the tetrahedral meshes had up to 6.2 million second-order cells with a resolution of at least 1.5 mm.

## Results

**Fig 5:**
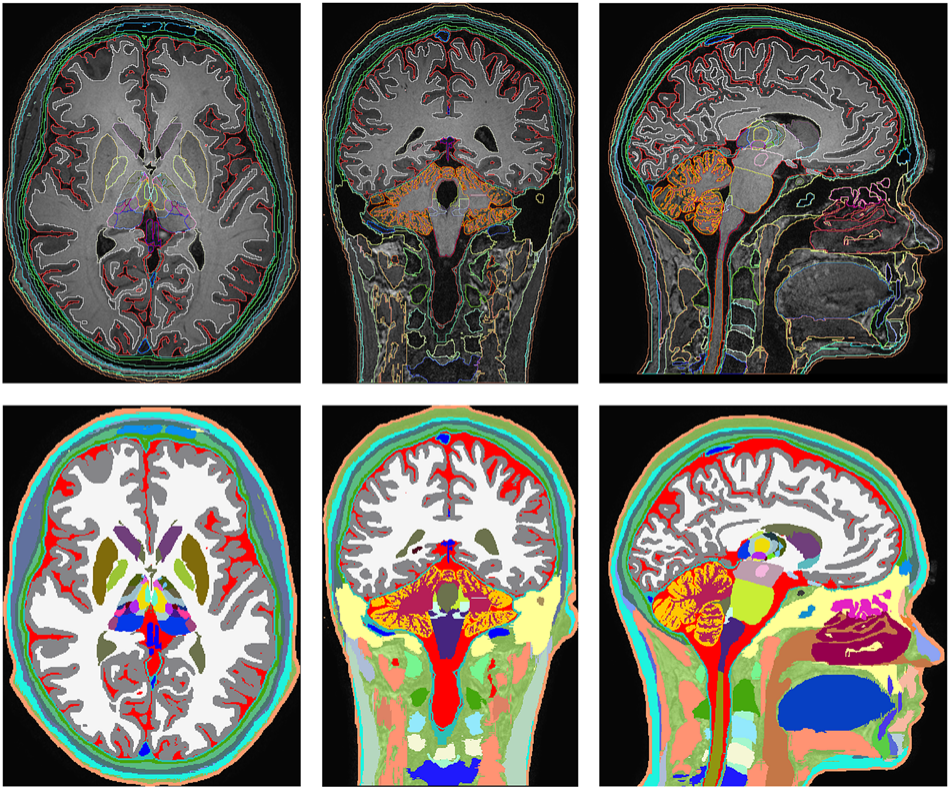
Final segmentation. Axial, coronal, and sagittal views of the outlines of the segmented head and neck structures (top row) and the color-coded label maps (bottom row).

**Fig 6:**
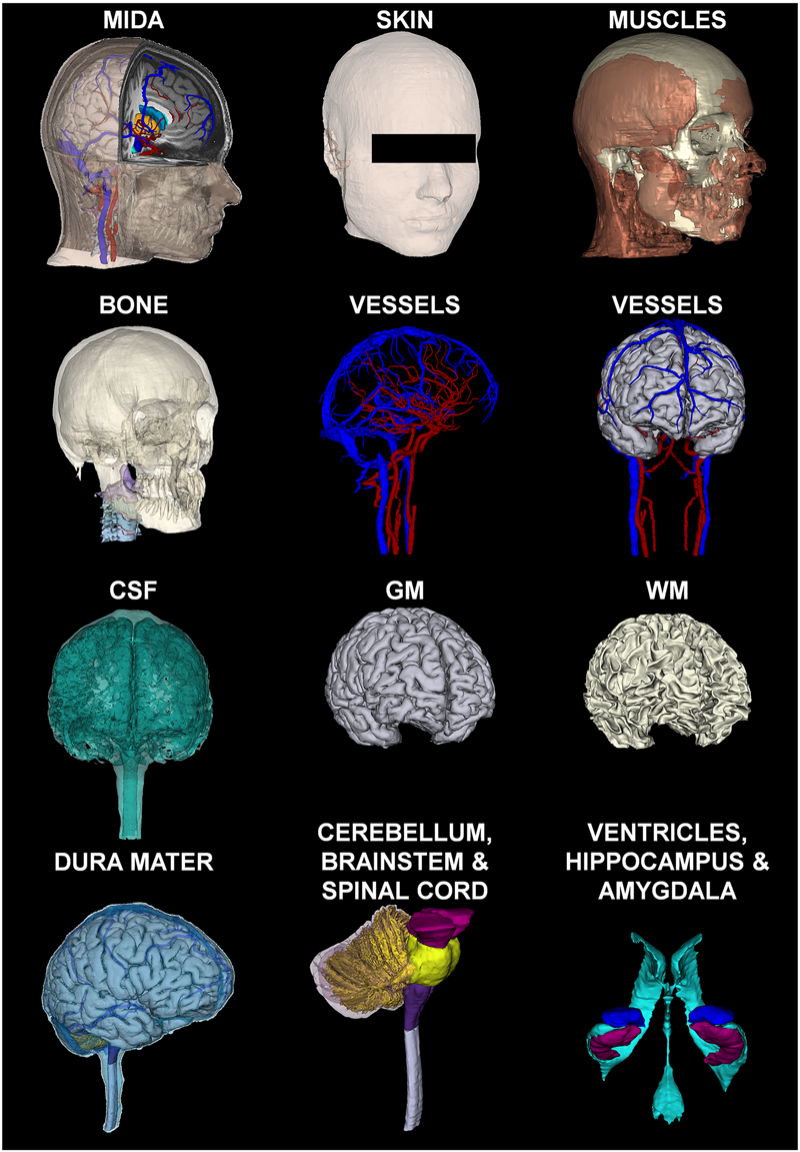
3D Surfaces. 3D reconstruction of a few representative structures of the head and neck. The muscles are shown with the skull structures. The vessels are shown both without and with the GM. The dura mater is shown on top of the brain and vessels.

The 500 μm isotropic resolution of the T1- and T2-weighted structural MRI and different contrast information they provided, together with the special slab for the ear and the eye, and the two MRA datasets allowed us to distinguish 153 anatomical structures in the head and neck. The list of segmented structures is reported in Tables 1 and 2. Fig 5 shows axial, coronal, and sagittal slices of the segmented MRIs (top) and the corresponding color-coded label maps (bottom). 3D surface reconstructions of representative structures of the head are provided in Fig 6.

**Table 1:** List of the segmented structures.

**Adipose Tissue**	**Ear Auditory Canal**	**Muscle—Platysma**
**Air Internal—Ethmoidal Sinus**	**Ear Auricular Cartilage (Pinna)**	**Muscle—Procerus**
**Air Internal—Frontal Sinus**	**Ear Cochlea**	**Muscle—Risorius**
**Air Internal—Mastoid**	**Ear Pharyngotympanic Tube**	**Muscle—Splenius Capitis**
**Air Internal—Maxillary Sinus**	**Ear Semicircular Canals**	**Muscle—Sternocleidomastoid**
**Air Internal—Nasal/Pharynx**	**Epidermis/Dermis**	**Muscle—Superior Oblique**
**Air Internal—Oral Cavity**	**Eye Aqueous**	**Muscle—Superior Rectus**
**Air Internal—Sphenoidal Sinus**	**Eye Cornea**	**Muscle—Temporalis/Temporoparietalis**
**Amygdala**	**Eye Lens**	**Muscle—Trapezius**
**Blood Arteries**	**Eye Retina/Choroid/Sclera**	**Muscle—Zygomaticus Major**
**Blood Veins**	**Eye Vitreous**	**Muscle—Zygomaticus Minor**
**Brain Gray Matter**	**Globus Pallidus**	**Nasal Septum (Cartilage)**
**Brain White Matter**	**Hippocampus**	**Nucleus Accumbens**
**Brainstem Medulla**	**Hyoid Bone**	**Optic Chiasm**
**Brainstem Midbrain**	**Hypophysis Or Pituitary Gland**	**Optic Tract**
**Brainstem Pons**	**Hypothalamus**	**Parotid Gland**
**Caudate Nucleus**	**Intervertebral Disc**	**Pineal Body**
**Cerebellum Gray Matter**	**Mammillary Body**	**Putamen**
**Cerebellum White Matter**	**Mandible**	**Skull**
**Cerebral Peduncles**	**Mucosa**	**Skull Diploë**
**Commissura (Anterior)**	**Muscle (General)**	**Skull Inner Table**
**Commissura (Posterior)**	**Muscle—Buccinator**	**Skull Outer Table**
**Cranial Nerve I—Olfactory**	**Muscle—Depressor Anguli Oris**	**Spinal Cord**
**Cranial Nerve II—Optic**	**Muscle—Depressor Labii**	**Subcutaneous Adipose Tissue**
**Cranial Nerve III—Oculomotor**	**Muscle—Inferior Oblique**	**Sublingual Gland**
**Cranial Nerve IV—Trochlear**	**Muscle—Inferior Rectus**	**Submandibular Gland**
**Cranial Nerve V—Trigeminal Nerve**	**Muscle—Lateral Pterygoid**	**Substantia Nigra**
**Cranial Nerve V2—Maxillary Division**	**Muscle—Lateral Rectus**	**Teeth**
**Cranial Nerve V3—Mandibular Division**	**Muscle—Levator Labii Superioris**	**Tendon—Galea Aponeurotica**
**Cranial Nerve VI—Abducens**	**Muscle—Levator Scapulae**	**Tendon—Temporalis**
**Cranial Nerve VII—Facial**	**Muscle—Masseter**	**Thalamus** ^**1**^
**Cranial Nerve VIII—Vestibulocochlear**	**Muscle—Medial Pterygoid**	**Tongue**
**Cranial Nerve IX—Glossopharyngeal**	**Muscle—Medial Rectus**	**Vertebra—C1 (atlas)**
**Cranial Nerve X—Vagus**	**Muscle—Mentalis**	**Vertebra—C2 (axis)**
**Cranial Nerve XI—Accessory**	**Muscle—Nasalis**	**Vertebra—C3**
**Cranial Nerve XII—Hypoglossal**	**Muscle—Occipitiofrontalis—Frontal Belly**	**Vertebra—C4**
**CSF General**	**Muscle—Occipitiofrontalis—Occipital Belly**	**Vertebra—C5**
**CSF Ventricles**	**Muscle—Orbicularis Oculi**	
**Dura**	**Muscle—Orbicularis Oris**	

^1^ Includes all the nuclei in Table 2, excluding the Red nucleus (RN) and the Subthalamic nucleus (STh)

**Table 2:** Nuclei obtained via atlas-based segmentation.

**Anterodorsal nucleus (AD)**	**Inferior Pulvinar (PuI)**
**Anteromedial nucleus (AM)**	**Lateral Pulvinar (PuL)**
**Anteroventral nucleus (AV)**	**Medial Pulvinar (PuM)**
**Central Lateral nucleus CL**	**Mammillothalamic Tract (mtt)**
**Centromedian nucleus (CM)**	**Paraventricular nuclei (Pv)**
**Central Medial nucleus (CeM)**	**Red nucleus (RN)**
**Habenular nucleus (Hb)**	**Subparafascicular nucleus (sPf)**
**Lateral Dorsal nucleus (LD)**	**Suprageniculate nucleus (SG)**
**Lateral Geniculate nucleus—Magnocellular part (LGNmc)**	**Subthalamic nucleus (STh)**
**Lateral Geniculate nucleus—Parvocellular part (LGNpc)**	**Ventral Anterior nucleus—Magnocellular part (VAmc)**
**Lateral Posterior nucleus (LP)**	**Ventral Anterior nucleus—Parvocellular part (VApc)**
**Limitans nucleus (Li)**	**Ventral Lateral Anterior nucleus (VLa)**
**Mediodorsal nucleus—Magnocellular part (MDmc)**	**Ventral Lateral—Posterior Dorsal part (VLpd)**
**Mediodorsal nucleus—Parvocellular part (MDpc)**	**Ventral Lateral—Posterior Ventral part (VLpv)**
**Medial Geniculate nucleus (MGN)**	**Ventral Medial nucleus (VM)**
**Medioventral nucleus (MV)**	**Ventral Posterior Inferior nucleus (VPI)**
**Parafascicular nucleus (Pf)**	**Ventral Posterior Lateral—Anterior part (VPLa)**
**Posterior nucleus (Po)**	**Ventral Posterior Lateral-Posterior part (VPLp)**
**Anterior Pulvinar (PuA)**	**Ventral Posterior Medial (VPM)**

**Fig 7:**
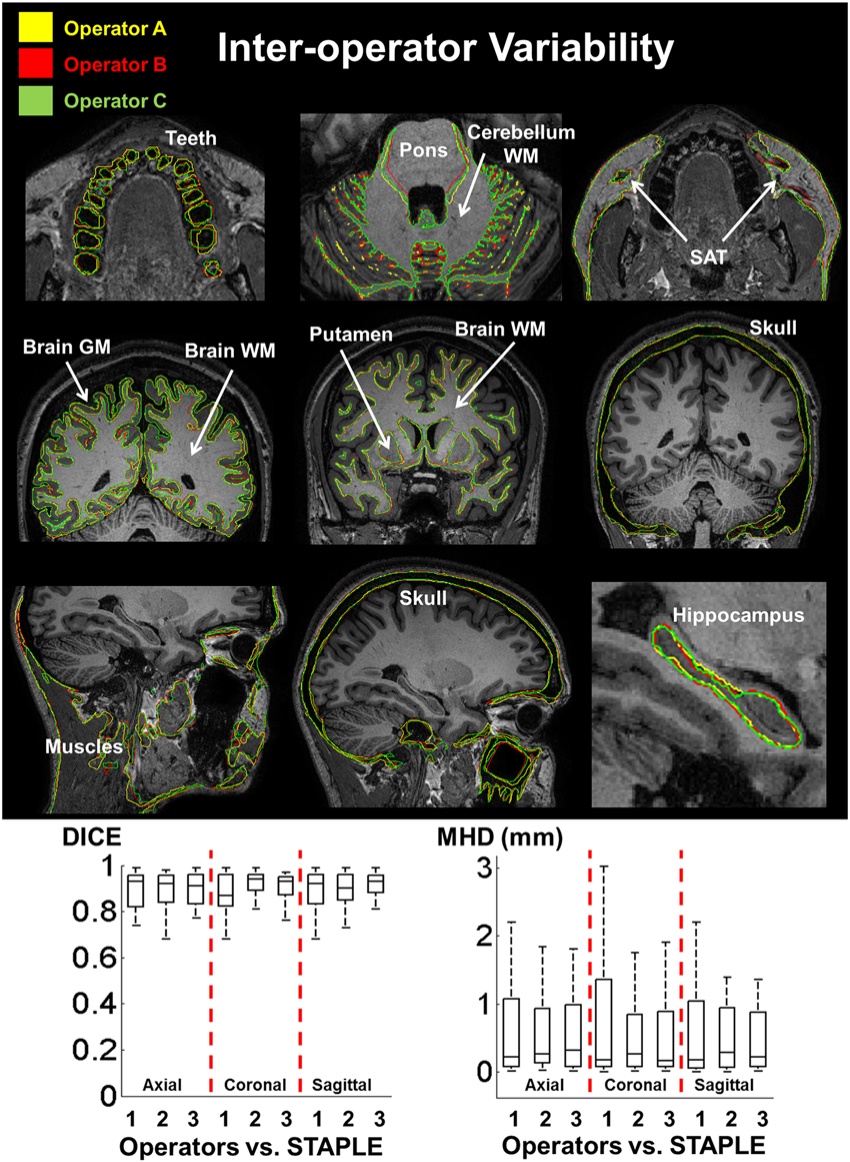
Inter-operator variability. Examples of segmentation variability among operators A (yellow), B (red), and C (green) for a few representative structures of the head. (Bottom) Box plots of the values of the Dice (*D*) and modified Hausdorff distance (*MHD*) for the 35 structures are included in the analysis. The variability was assessed by comparing the segmentations of the operators with a consensus ground truth obtained using the STAPLE algorithm.

Inter-operator variability indexes are summarized in Tables 3–5 for the axial, coronal, and sagittal slices respectively. Fig 7 shows an example of the segmentations of operators A (yellow), B (red), and C (green) for a few representative structures of the head. Box plots of the values of the Dice index (*D*) and modified Hausdorff distance (*MHD*) for the 35 structures included in the analysis are given at the bottom of Fig 7. The inter-operator variability was ranked as *D* = 0.89 ± 0.09 (mean ± SD) and *MHD* = 0.56 mm ± 0.62 mm across operators and anatomical structures. The highest matching (*D* values >0.95 and *MHD <* 0.1mm for all the comparisons) was reported for the spinal cord and the putamen. Lower matching (*D* values < 0.85 and *MHD>*0.8 mm) was found for structures like the adipose tissue and the general CSF fluid which are fragmentary filling material, and for thin layers like the dura, the skin, and the galea aponeurotica. For each operator, Lilliefors’ test was used to determine whether the *D* indexes and the *MHD* across structures were normally distributed. Accordingly, the non-parametric Kruskal-Wallis test of variance was used for groups’ comparison. The analysis suggested no significant inter-observer variability among operators in terms of *D* indexes (p-value = 0.61) and *MHD* values (p-value = 0.96) with 95% confidence.

**Table 3:** Inter-operator variability across structures assessed on the axial slices.

Axial Segmentation	DICE			Modified Hausdorff distance (mm)		
	1 vs. GT	2 vs. GT	3 vs. GT	1 vs. GT	2 vs. GT	3 vs. GT
**Adipose Tissue**	0.77	0.84	0.84	2.20	1.84	1.81
**Brain Gray Matter**	0.82	0.96	0.95	0.84	0.43	0.48
**Brain White Matter**	0.93	0.95	0.94	0.37	0.26	0.33
**Brainstem Pons**	0.99	0.92	0.96	0.03	0.14	0.07
**Cerebellum Gray Matter**	0.93	0.93	0.98	0.75	0.75	0.32
**Cerebellum White Matter**	0.94	0.97	0.91	0.48	0.32	0.63
**CSF General**	0.84	0.83	0.78	1.06	1.06	1.18
**CSF Ventricles**	0.99	0.90	0.86	0.01	0.05	0.06
**Dura**	0.77	0.68	0.77	1.07	1.24	1.07
**Ear Auricular Cartilage (Pinna)**	0.74	0.80	0.84	0.15	0.13	0.12
**Epidermis/Dermis**	0.76	0.82	0.80	1.65	1.43	1.52
**Eye Aqueous**	0.81	0.79	0.82	0.13	0.13	0.13
**Eye Lens**	0.94	0.92	0.77	0.03	0.02	0.06
**Eye Vitreous**	0.90	0.94	0.99	0.16	0.15	0.01
**Mandible**	0.96	0.97	0.93	0.08	0.06	0.11
**Muscle (General)**	0.88	0.98	0.91	2.05	0.91	1.78
**Parotid Gland**	0.96	0.96	0.96	0.17	0.18	0.18
**Skull**	0.90	0.88	0.85	1.26	1.37	1.61
**Spinal Cord**	**0.98**	**0.96**	**0.99**	**0.01**	**0.02**	**0.01**
**Subcutaneous Adipose Tissue (SAT)**	0.82	0.94	0.96	2.07	0.94	0.72
**Teeth**	0.93	0.84	0.83	0.22	0.38	0.38
**Tongue**	0.99	0.94	0.93	0.07	0.19	0.22
**Vertebrae**	0.95	0.91	0.96	0.07	0.11	0.06

**Table 4:** Inter-operator variability across structures assessed on the coronal slices.

Coronal Segmentation	DICE			Modified Hausdorff distance (mm)		
	1 vs. GT	2 vs. GT	3 vs. GT	1 vs. GT	2 vs. GT	3 vs. GT
**Adipose Tissue**	0.60	0.89	0.89	3.02	1.75	1.90
**Air Internal—Nasal**	0.98	0.91	0.93	0.07	0.17	0.08
**Air Internal—Pharynx**	0.73	0.79	0.90	0.17	0.14	0.07
**Air Internal—Sphenoidal Sinus**	0.87	0.92	0.76	0.07	0.05	0.08
**Brain Gray Matter**	0.85	0.96	0.95	2.09	1.08	1.14
**Brain White Matter**	0.96	0.98	0.97	1.04	0.80	0.89
**Brainstem Medulla**	0.95	0.95	0.87	0.07	0.02	0.06
**Caudate Nucleus**	0.87	0.96	0.97	0.07	0.04	0.04
**Cerebellum Gray Matter**	0.83	0.87	0.97	0.83	0.72	0.36
**Cerebellum White Matter**	0.99	0.94	0.91	0.10	0.49	0.62
**CSF General**	0.82	0.90	0.86	1.58	1.19	1.43
**CSF Ventricles**	0.89	0.95	0.96	0.14	0.11	0.07
**Dura**	0.84	0.56	0.68	1.13	1.69	1.50
**Epidermis/Dermis**	0.68	0.91	0.84	1.77	0.91	1.30
**Hypophysis or Pituitary gland**	0.84	0.99	0.86	0.04	0.01	0.03
**Mandible**	0.97	0.95	0.95	0.08	0.09	0.11
**Muscle (General)**	0.82	0.95	0.95	2.24	1.30	1.31
**Optic Tract**	0.98	0.92	0.95	0.00	0.01	0.01
**Parotid Gland**	0.93	0.81	0.90	0.04	0.07	0.04
**Putamen**	**0.96**	**0.96**	**0.95**	**0.05**	**0.07**	**0.07**
**Skull**	0.87	0.97	0.97	1.43	0.72	0.75
**Spinal Cord**	**0.98**	**0.98**	**0.95**	**0.03**	**0.09**	**0.09**
**Subcutaneous Adipose Tissue (SAT)**	0.73	0.92	0.92	1.61	0.85	0.89
**Submandibular Gland**	0.96	0.97	0.96	0.13	0.10	0.15
**Tendon—Galea Aponeurotica**	0.78	0.88	0.83	0.57	0.43	0.49
**Tongue**	0.93	0.97	0.95	0.24	0.26	0.16
**Vertebrae**	0.92	0.86	0.88	0.32	0.43	0.41

**Table 5:** Inter-operator variability across structures assessed on the sagittal slices.

Sagittal Segmentation	DICE			Modified Hausdorff distance (mm)		
	1 vs. GT	2 vs. GT	3 vs. GT	1 vs. GT	2 vs. GT	3 vs. GT
**Adipose Tissue**	0.79	0.94	0.93	2.20	1.26	1.28
**Amygdala**	0.95	0.77	0.95	0.01	0.03	0.01
**Brain Gray Matter**	0.95	0.96	0.96	1.06	0.97	0.94
**Brain White Matter**	0.97	0.98	0.97	0.88	0.72	0.77
**Cerebellum Gray Matter**	0.98	0.86	0.93	0.18	0.47	0.34
**Cerebellum White Matter**	0.99	0.81	0.89	0.04	0.28	0.22
**CSF General**	0.82	0.86	0.90	1.17	1.04	0.88
**CSF Ventricles**	0.84	0.97	0.89	0.08	0.04	0.06
**Dura**	0.84	0.54	0.70	0.84	1.28	1.09
**Epidermis/Dermis**	0.68	0.86	0.90	1.51	1.03	0.85
**Eye Vitreous Humor**	0.85	0.98	0.99	0.09	0.04	0.03
**Hippocampus**	0.92	0.94	0.94	0.06	0.05	0.05
**Mandible**	0.96	0.93	0.91	0.13	0.19	0.20
**Muscle (General)**	0.91	0.99	0.94	1.63	0.62	1.36
**Optic Tract**	0.99	0.83	0.72	0.01	0.01	0.02
**Parotid Gland**	0.96	0.86	0.81	0.05	0.12	0.12
**Putamen**	**0.95**	**0.96**	**0.96**	**0.05**	**0.06**	**0.07**
**Skull**	0.83	0.96	0.96	1.60	0.86	0.82
**Subcutaneous Adipose Tissue (SAT)**	0.92	0.85	0.88	0.99	1.39	1.32
**Submandibular Gland**	0.96	0.96	0.96	0.17	0.17	0.18
**Teeth**	0.70	0.90	0.94	0.17	0.12	0.09
**Tendon—Galea Aponeurotica**	0.52	0.85	0.87	0.66	0.43	0.42
**Vertebrae**	0.91	0.73	0.67	0.03	0.07	0.09

**Fig 8:**
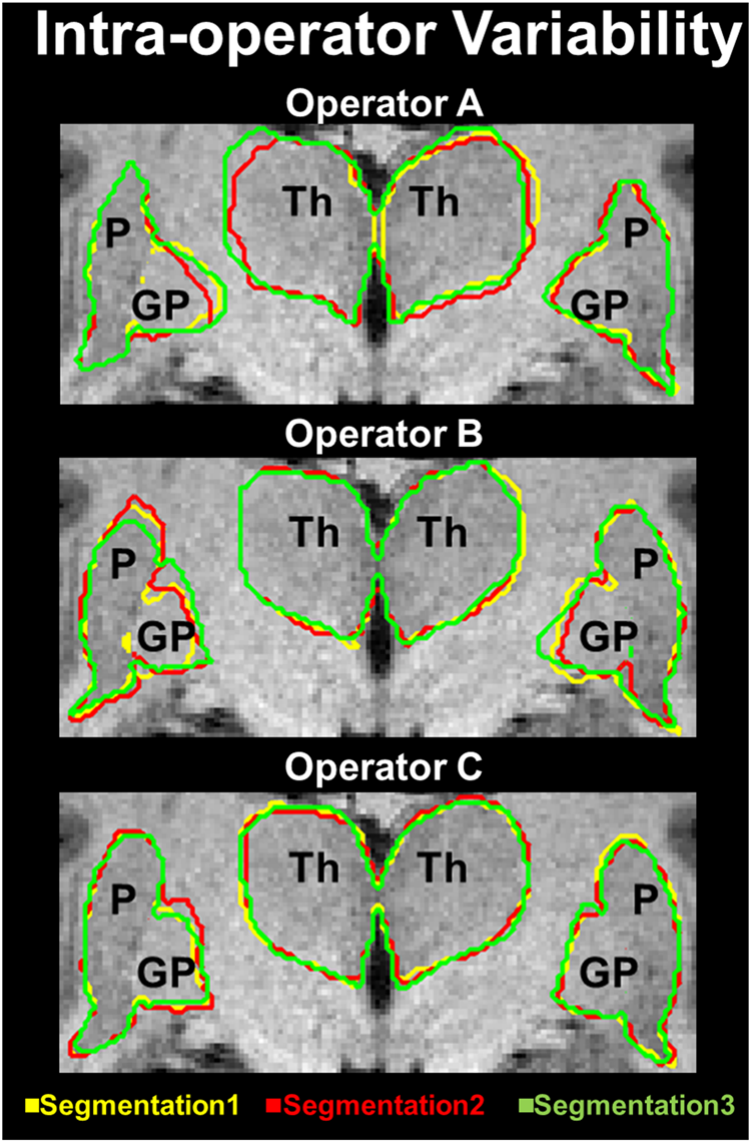
Intra-operator variability. Segmentation1 (yellow), Segmentation2 (red), and Segmentation3 (green) are provided for operators A, B and C on the top, middle, and bottom of the figure, respectively. The intra-operator variability was quantified asking each operator to repeat the segmentation of three selected structures (i.e., the globus pallidus, putamen, and thalamus) three times and measuring the similarity between the outlines performed by each user on different days and a consensus ground truth obtained using the STAPLE algorithm.

The intra-operator variability (Fig 8) was calculated for each operator by comparing the three repeated segmentations against the STAPLE ground truth, e.g., for operator A: Segmentation1 vs. GT, Segmentation2 vs. GT, and Segmentation3 vs. GT. We obtained *D* indexes of 0.98 ± 0.02, 0.96 ± 0.02, and 0.97 ± 0.01 and MHD values of 2.02 mm ± 1.51 mm, 2.67 mm ± 0.48 mm, and 2.46 mm ± 0.43 mm for operators A, B, and C, respectively.

**Fig 9:**
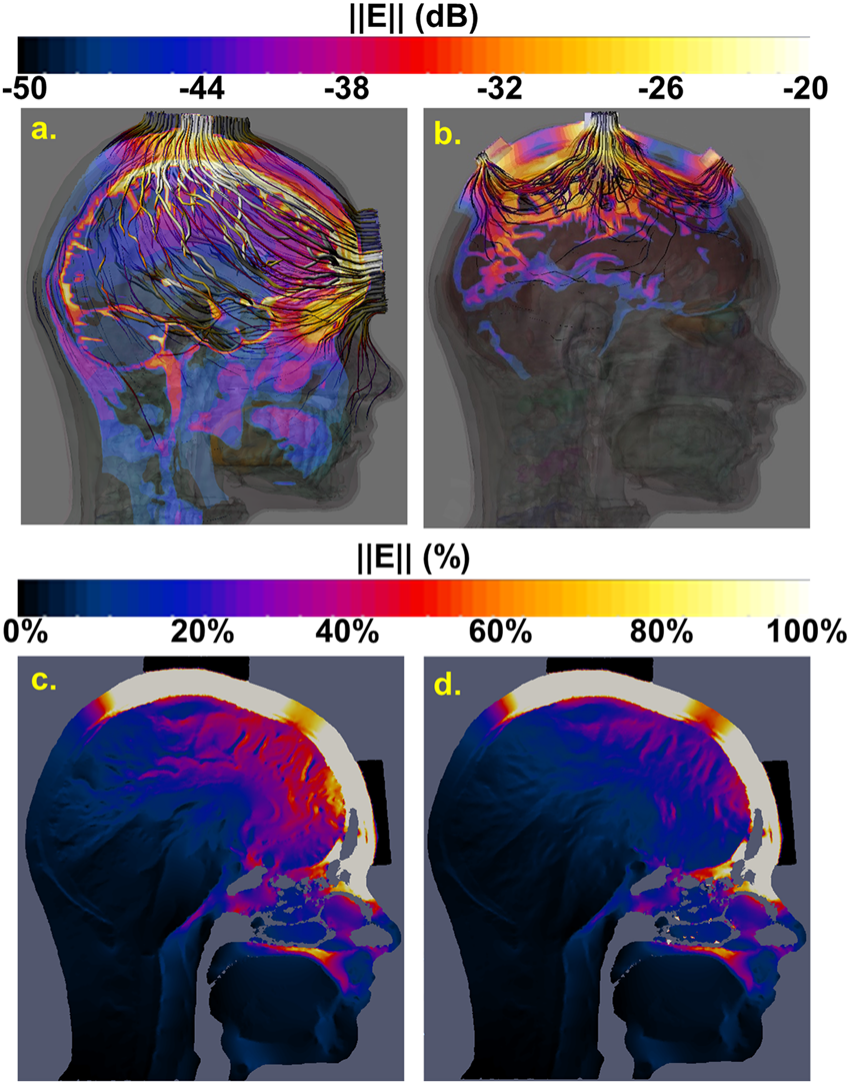
tACS modeling (Top). Comparison of two montages of stimulus electrode configurations. Sagittal view of the simulated magnitude of the electric field through the eye and of the current density stream lines for the (Fpz-Cz) montage (a) and the Cz-(Fz, C3, C4, Pz) montage (b). The first montage generated higher currents through the eye globe, which includes the retina, compared to the latter montage. (Bottom) Comparison of tissue-specific scalar vs. image-based tensorial electrical properties. Simulated magnitude of the electric field resulting from the (Fpz-Cz) montage tACS based on tissue-specific scalar electrical conductivity values (c) and DTI-based anisotropic values in brain tissues (d). DTI-based conductivity reduces the predicted field strength in the brain. The scale was set to dB (top) and linear (bottom) for visualization purposes.

A case-study based on transcranial alternating current stimulation was modeled using different numerical methods, tetrahedral elements vs. rectilinear voxels discretization approaches, and tissue specific scalar electrical conductivity vs. DTI-based anisotropic electrical conductivity approaches for tissue property assignment. Fig 9a and 9b show a comparison of the current distributions for the two electrode setups used for the stimulation. The Fpz-Cz electrode placement resulted in higher currents through the retina [76] while with Cz-(Fz, C3, C4, Pz), exposure of the retina was mostly avoided. The high resolution structured mesh simulations showed results similar to those of the coarser adaptive, conformal, unstructured mesh simulations, as long as the unstructured mesh was fine enough to resolve the skin, the skull layers, and the dura. When comparing a simulation based on scalar, tissue-wise homogeneous tissue properties to one in which inhomogeneous, anisotropic, image-based properties were used, similar field distributions were obtained, but the electric field inside the brain was predicted to be about 25% weaker when the image-derived anisotropic conductivity was considered (Fig 9c and 9d), whereas the effect on the current distribution was less pronounced.

## Discussion

Early anatomical models of the human body were mostly represented by homogeneous or multi-layer geometric volumes, such as slabs, cylinders, and spheres. Examples of such simplified models can be found for radiation dosimetry applications [80,81], electromagnetic analysis [82–85], and biomechanics [86,87]. Simplified geometries can provide only a surrogate representation of the organs and do not take into account the topological and morphological complexity of the human anatomy. The advent of new computing technologies, such as graphics processing units (GPU) and high performance computing (HPC), and the increasing computing power (i.e., orders of magnitude every year) have catalyzed the proliferation of image-based anthropomorphic models. The field of electromagnetic dosimetry was one of the first to adopt image-based models because they offer detailed information about the complex field distributions inside the human body under very controlled conditions. Indeed, evaluations based on image-based anatomical models offer information about the variability of electromagnetic fields as a function of the anatomical region under investigation and varying tissue properties. Although high complexity is not always a must in computational modeling, a high resolution model, as MIDA, offers the user the flexibility of choosing the level of model accuracy accordingly on available computational resources, as well as the nature of the problem being investigated [88]. The MIDA model was created primarily as a tool to simulate the interactions of tissues with electromagnetic fields generated by medical devices as well as for electromagnetic dosimetry. DTI have also been integrated to investigate anisotropic tissue conductivity in the brain. In addition, the large number of distinguished muscles and bones make MIDA interesting for biomechanical applications, while the distinction of skull layers and detailed deep brain structures may be valuable for transcranial focused ultrasound modeling and applications. Ionizing radiation exposure assessment benefits from the large number of distinguished structures including salivary glands.

The image-based modeling framework requires three fundamental steps, namely 1) image acquisition, 2) image segmentation and labeling, and 3) surface mesh generation.

### Image Acquisition

The imaging acquisition protocol plays a key role defining the achievable level of the anatomical detail in the model. In this study, we used a comprehensive multi-modal imaging method to resolve the different structures of the head and neck in detail. The model was obtained by integrating different MRI modalities, the parameters of which were tailored to enhance the signals of specific tissues.

**Fig 10:**
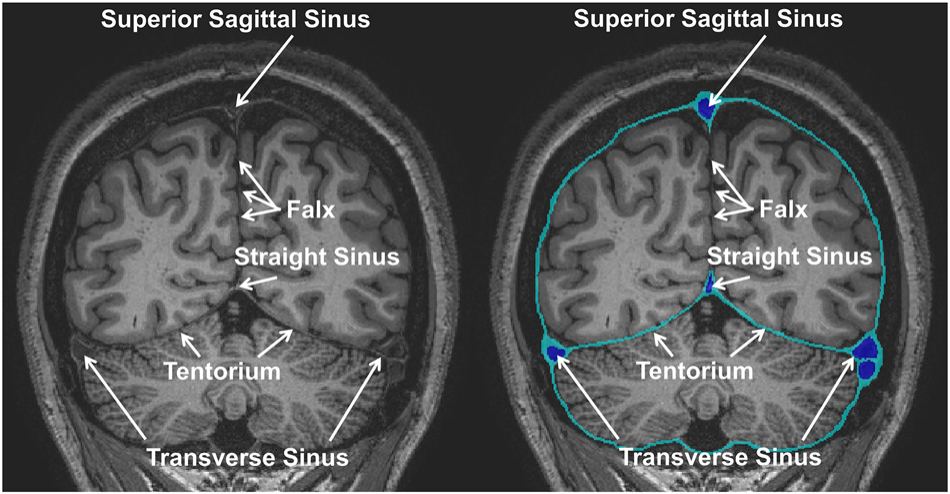
Segmentation of the dura mater. Coronal T1-weighted MRI (left) and outlines of the dura mater (right) with the principal dural reflections and sinuses. The falx cerebri, which separates the two cerebral hemispheres, was only partially visible on the images and was not segmented. The tentorium cerebelli was manually segmented. The superior sagittal sinus, transverse sinuses, and straight sinus—found along the attached edge of the falx, the line of attachment of the tentorium, and the line of attachment of the falx to the tentorium, respectively—were modeled as 500 μm thick outer layers enveloping the large venous vessels visible in the MRA dataset.

**Fig 11:**
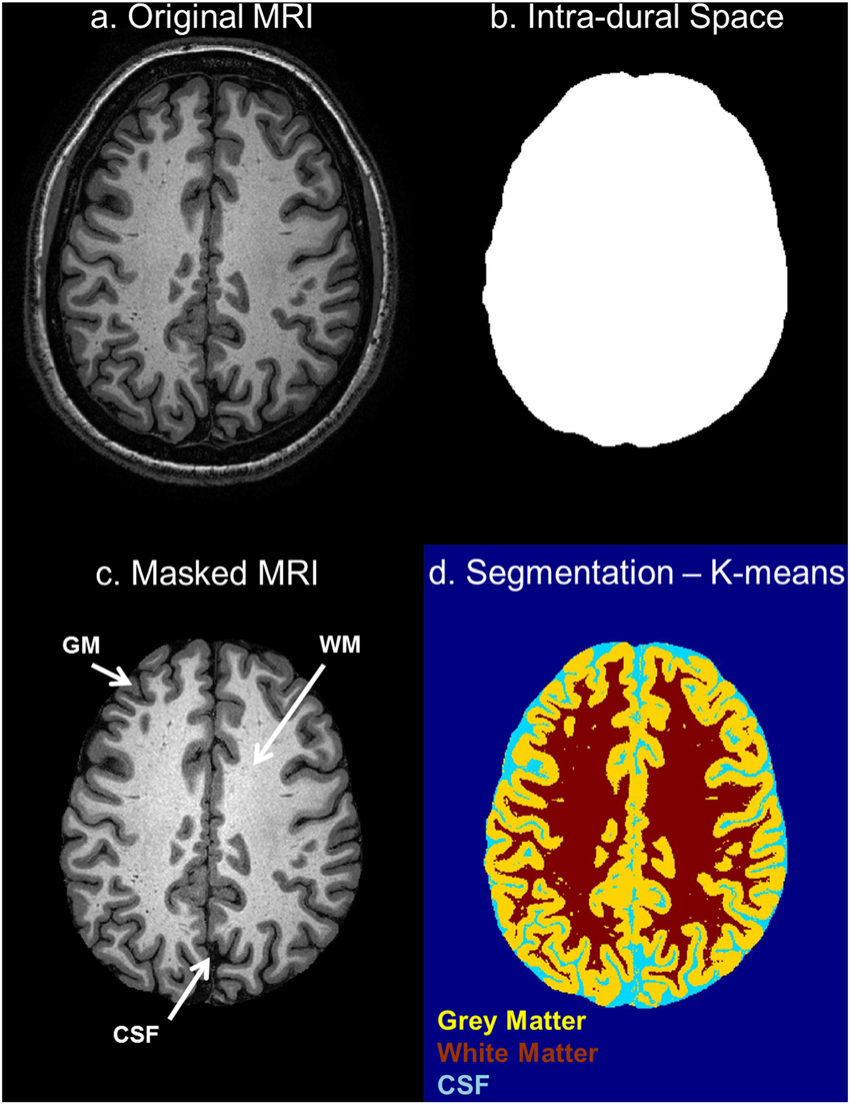
Segmentation of the cerebrum. (a) Original axial T1-weighted MRI, (b) intra-dural space obtained as the space surrounded by the previously segmented dura mater, (c) masked T1-weighted MRI, and (d) the image resulting from application of the *k*-means algorithm.

**Fig 12:**
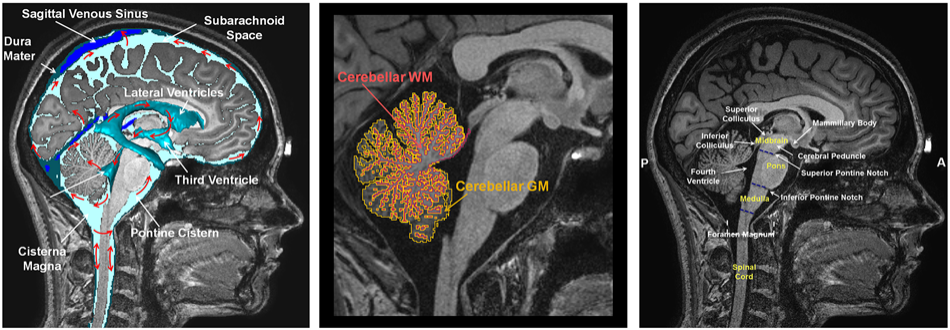
Segmentation of the brainstem, cerebellum, and CSF. Mid-coronal slice of the T1-weighted MRI (left) showing the CSF circulation (red arrows). Magnified view of the mid-coronal slice of the cerebellum (middle), showing the segmentation of the cerebellar GM (yellow) and WM (red). Mid-coronal slice of the brainstem and spinal cord (right), showing the landmarks used to subdivide the brainstem into its constituent substructures, i.e., the midbrain, pons, and medulla, and to separate the brainstem from the spinal cord.

**Fig 13:**
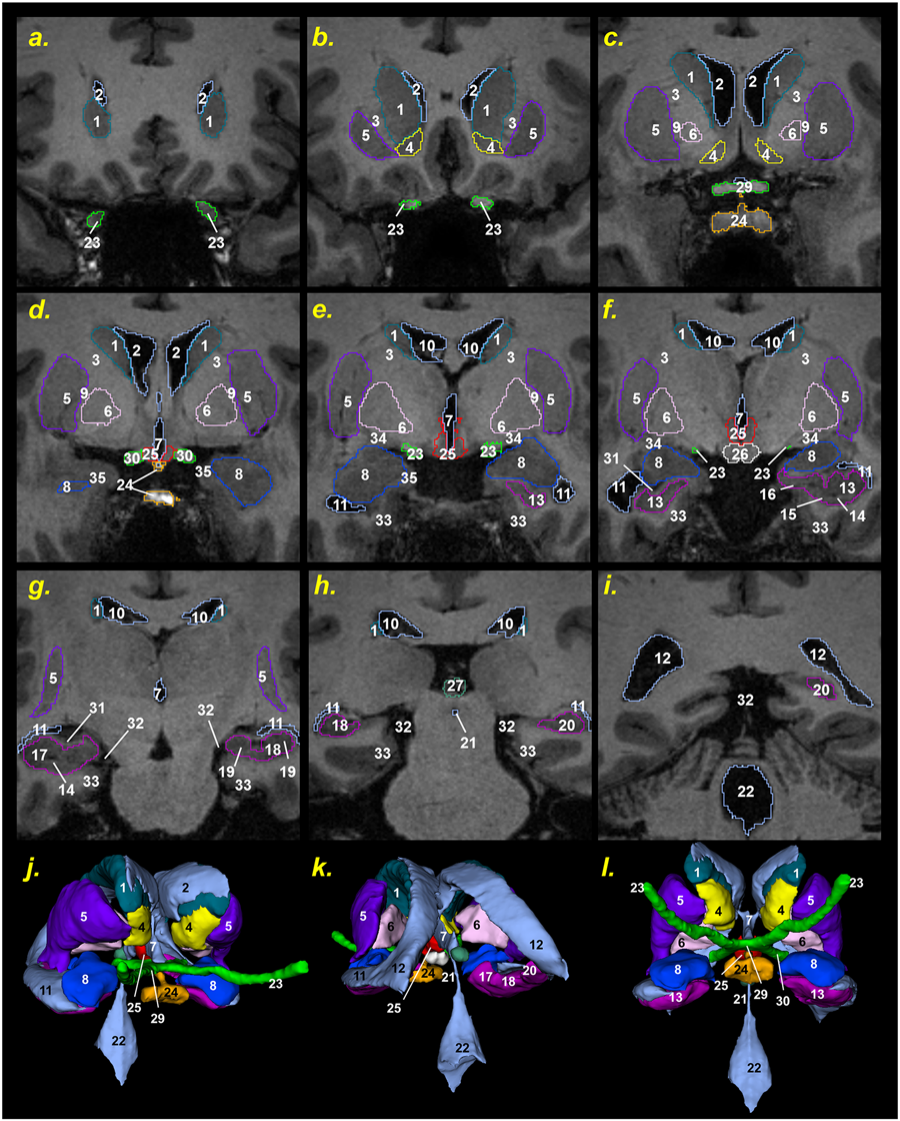
Segmentation of deep brain structures. Coronal slices of the deep brain structures from anterior to posterior (from a to i). Legend of the structures: 1 Caudate nucleus; 2 Lateral ventricles (anterior horn); 3 Internal capsule; 4 Nucleus accumbens; 5 Putamen; 6 Pallidum; 7 Third ventricle; 8 Amygdala; 9 External medullary lamina; 10 Lateral ventricles (body); 11 Lateral ventricles (inferior horn); 12 Lateral ventricles (collateral trigone); 13 Hippocampus (head); 14 Uncal sulcus; 15 Subiculum; 16 Uncinate gyrus; 17 Hippocampus (head)—gyrus dentatus; 18 Hippocampus (body)—gyrus dentatus; 19 Cornus ammonis; 20 Hippocampus (tail)—gyrus dentatus; 21 Cerebral aqueduct; 22 Fourth ventricle; 23 Optic nerve; 24 Pituitary gland and pituitary stalk or hypophysis and infundibulum; 25 Hypothalamus; 26 Mammillary body; 27 Pineal gland; 28 Anterior commissure; 29 Optic chiasm; 30 Optic tract; 31 Alveus; 32 Cisterna ambiens; 33 Parahippocampal gyrus; 34 Ansa peduncularis; 35 Entorhinal cortex. Right anterior oblique (j), left posterior oblique (k) and ventral caudal (l) view of the 3D reconstruction of the deep brain structures.

**Fig 14:**
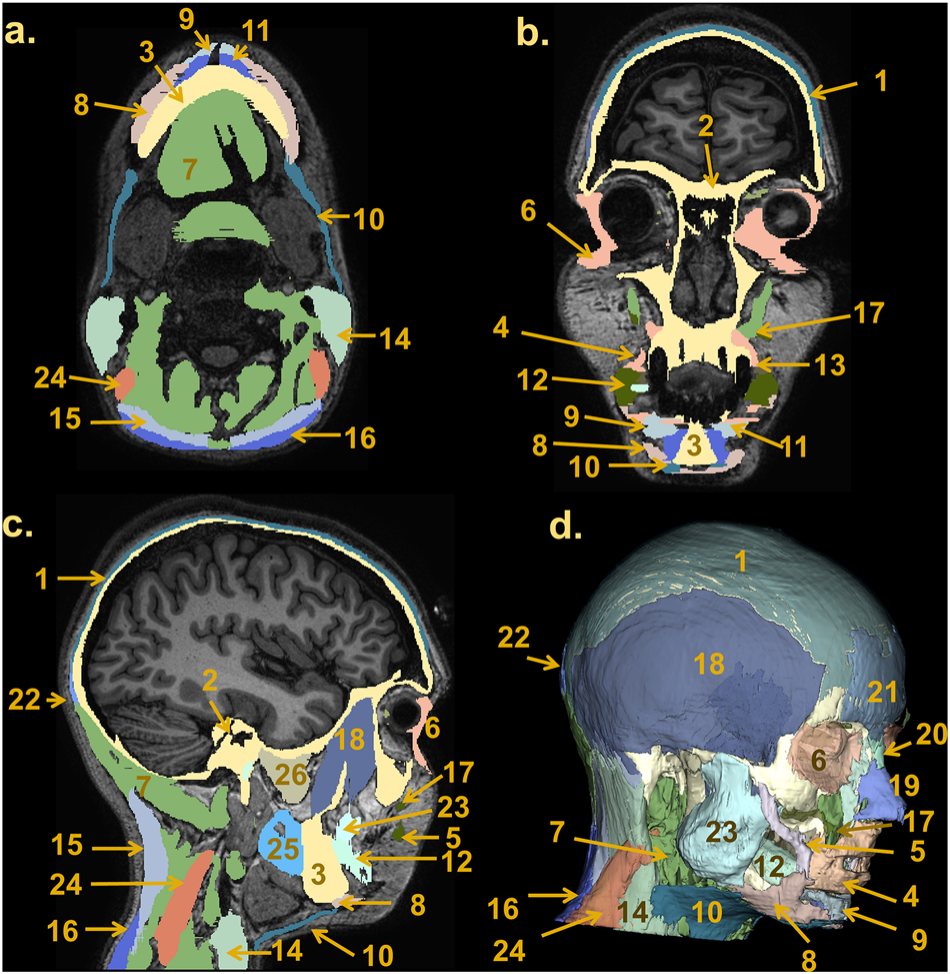
Segmentation of muscles. Axial (a), coronal (b) and sagittal (c) views of the individual and general muscle labels in the T1-weighted dataset. (d) Right anterior oblique view of the 3D reconstruction of the muscles. Legend of the structures: 1 Galea aponeurotica (tendon), 2 Skull, 3 Mandible, 4 Orbicularis oris, 5 Zygomaticus major, 6 Orbicularis oculi, 7 Muscles (general), 8 Depressor anguli oris, 9 Depressor labii inferioris, 10 Platysma, 11 Mentalis, 12 Buccinator, 13 Risorius, 14 Sternocleidomastoid, 15 Splenius capitis, 16 Trapezius, 17 Zygomaticus minor, 18 Temporalis & temporoparietalis, 19 Nasalis, 20 Procerus, 21 Occipitofrontalis frontal belly, 22 Occipitofrontalis occipital belly, 23 Masseter, 24 Levator scapulae, 25 Medial pterygoid, 26 Lateral pterygoid.

**Fig 15:**
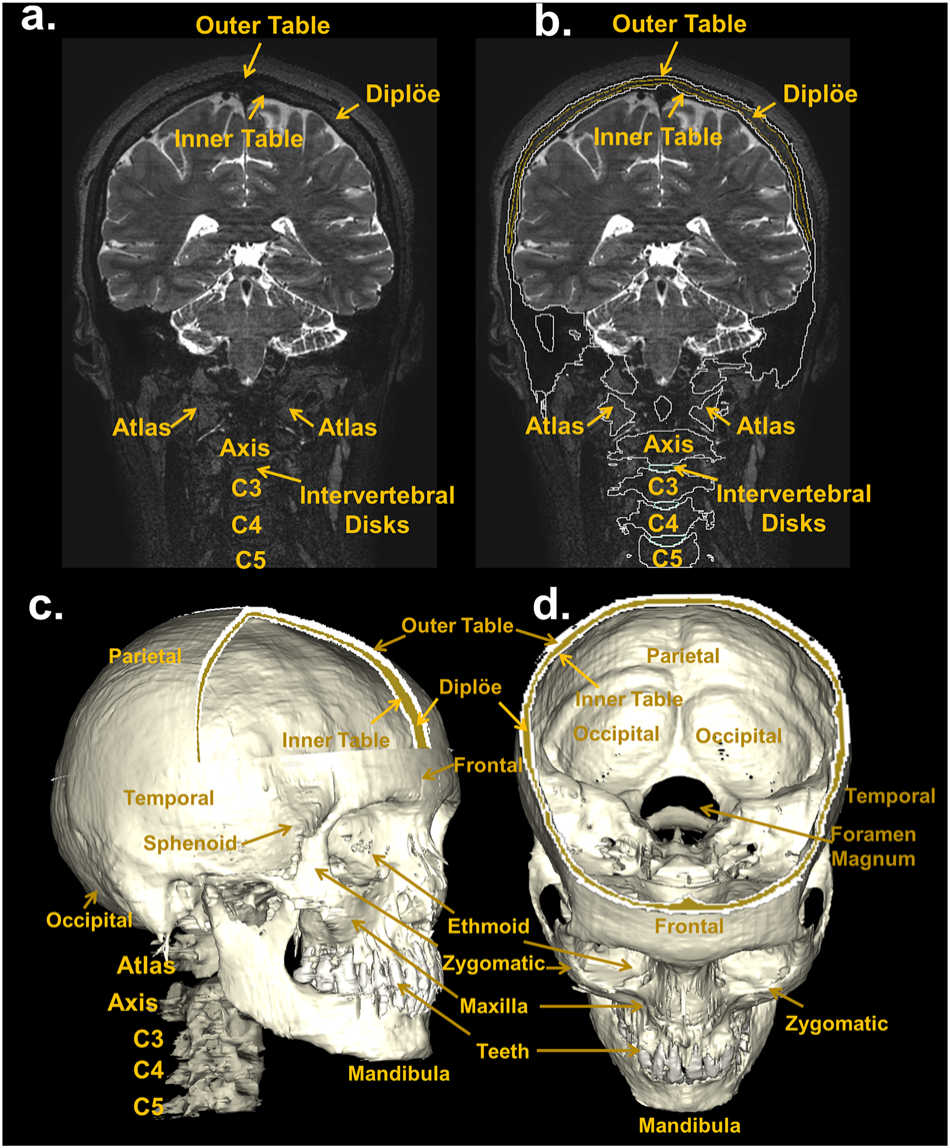
Segmentation of the bone. Top: coronal view of a T2-weighted MRI slice (a) with the skull, vertebrae, and intervertebral disks outlined (b). The T2-weighted MRI, in which the intensity of the cancellous bone inside the diploë is enhanced compared to that of the cortical bones of the inner and outer tables made the subdivision of the skull into the main three layers, i.e., outer table (white outline), diploë (yellow outline), and inner table (white outline) possible. Bottom: 3D reconstruction of the skull, vertebrae, and intervertebral disks (c and d).

The T1-weighted MRI images, optimized to enhance the contrast between gray matter (GM) and WM, allowed us to outline the dura mater with high precision (Fig 10)—with its principal reflections and sinuses—and the sulci of the cerebral cortex (Fig 11), which are CSF-filled fissures in the brain. It was also possible to discriminate the CSF-filled brain structures, including the subarachnoid space, the ventricular system, and the cisterns, and to reconstruct the CSF circulation (Fig 12, left). The improved contrast and resolution of the available T1 images allowed most of the GM and the WM present both in the core and in the fine-grained folia of the cerebellum to be captured and outlined (Fig 12, middle). Conversely, the majority of the models reported in the literature defined the cerebellum as a single structure without discerning between cerebellar GM and WM. Only a few models [41,89] have included the segmentation of the medullary core, which includes the central WM mass and the cerebellar nuclei located between the hemispheres of the cerebellum. Finally, a number of subcortical structures discernable on the images were included in the segmentation (see S1 Appendix, section 11). The discrimination of these brain landmarks was crucial for guiding the segmentation of the brainstem and spinal cord (Fig 12, right). The result of the segmentation of the major subcortical structures is provided on several coronal slices at different levels from most anterior (a) to most posterior (i) in Fig 13. The quality of the T1- and T2-weighted MRIs was also essential for the visualization of the muscles—which were clearly visible and separable (Fig 14)—and for outlining the bones and vertebrae. The subdivision of the skull in its main three layers, i.e., outer table, diploë, and inner table, was possible with the T2-weighted MRI, which provides enhanced intensity of the cancellous bone inside the diploë compared to cortical bone of the inner and outer tables (Fig 15). Distinguishing these skull layers can be important, e.g., for transcranial focused ultrasound (FUS) modeling. Because of the detailed segmentation of the muscles and skull, the model is also proposed as an investigative tool for biomechanical applications, such as in the analysis of head and neck injury.

**Fig 16:**
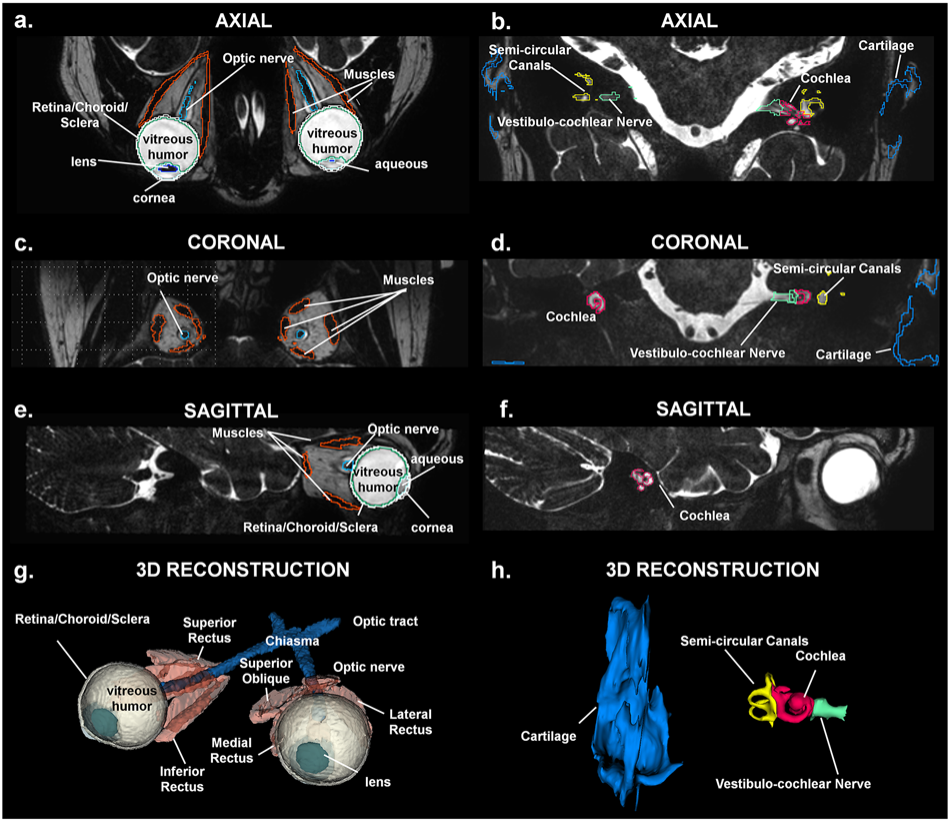
Segmentation of the eye and ear. T2 weighted axial, coronal and sagittal slab for the eye (left) and ear (right). The improved contrast allows for delineation of several thin substructures of the eye—e.g., optic nerve and lens—and ear—e.g., cochlea, semi-circular canals, and vestibulocochlear nerve—and the surrounding fat and fluids. 3D reconstruction (bottom) of the eye (left) and the ear (right) substructures.

**Fig 17:**
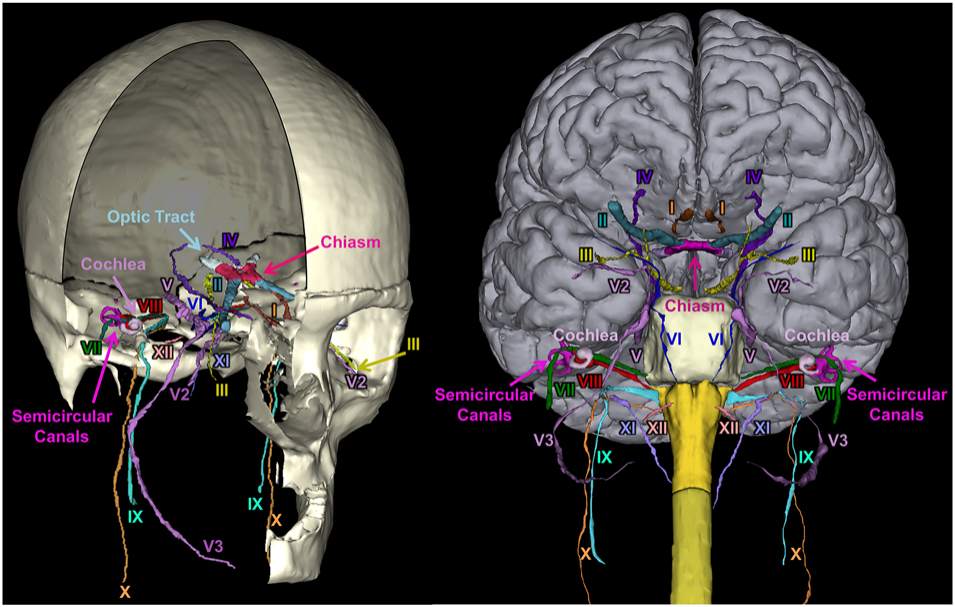
Segmentation of the nerves. 3D reconstruction of the 12 cranial nerves. Legend of the structures: Cranial Nerve I: Olfactory, Cranial Nerve II: Optic, Cranial Nerve III: Oculomotor, Cranial Nerve IV: Trochlear, Cranial Nerve V: Trigeminal, Cranial Nerve V2: Trigeminal Maxillary Division, Cranial Nerve V3: Trigeminal Mandibular Division, Cranial Nerve VI: Abducens, Cranial Nerve VII: Facial, Cranial Nerve VIII: Vestibulocochlear, Cranial Nerve IX: Glossopharyngeal, Cranial Nerve X: Vagus, Cranial Nerve XI: Accessory, Cranial Nerve XII: Hypoglossal.

The very long echo time (TE) used for the heavily T2-weighted thin slab for the ear and eye (Fig 16) provided a high-intensity signal for CSF, an intermediate-intensity signal for fat, and a low-intensity signal for other tissues, e.g., nerves, muscles, and lens. Data obtained with this sequence allowed for improved contrast and for segmentation of some of the cranial nerves (Fig 17), several substructures of the eye, the cochlea, and the semi-circular canals of the inner ear with its surrounding fluids and fatty tissues.

**Fig 18:**
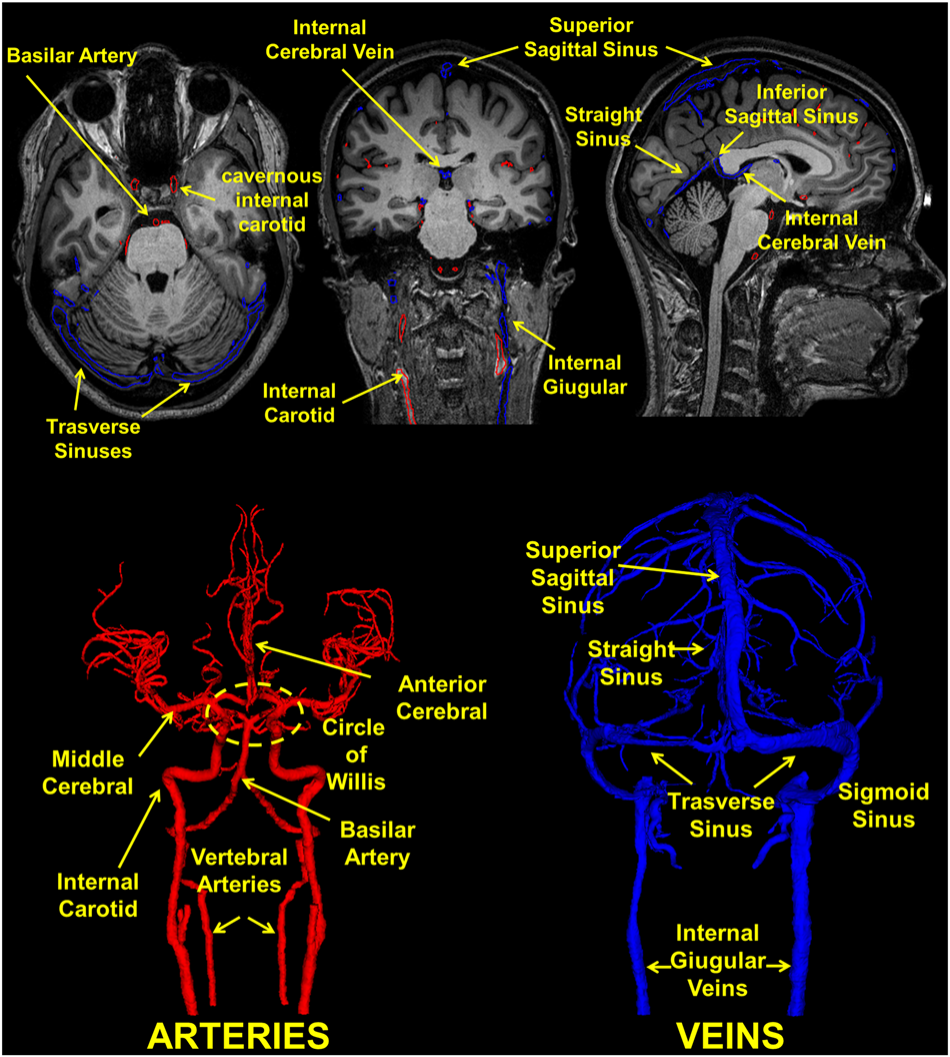
Segmentation of the vasculature. (Top) T1 weighted axial, coronal and sagittal view with the outline of the arteries (red) and veins (blue) and (Bottom) 3D reconstruction of the vessels. The arrows highlight the anterior and middle cerebral arteries, the basilar artery and the internal carotid which converge at the center to form the circle of Willis that supplies blood to the brain. Furthermore, the major veins are shown, including the dural venous sinuses running within the layers of the dura mater.

The MRA sequences provided better visualization of the vasculature compared to T1- and T2-weighted MRIs by suppressing the signal from static tissue and enhancing the signal coming exclusively from flowing blood. The TOF images encode flow direction information and are sensitive to the slicing direction and order. Therefore, these were optimized to highlight blood flowing in the cranial direction, which mostly results in arteries being visible (Fig 18, left). The PCA can be tweaked to particularly highlight specific velocity intervals. The velocity window has been chosen such that the high velocities observed in larger arteries are not highlighted, and images show mostly veins (Fig 18, right). While neither PCA nor TOF perfectly isolate arteries from veins, having both MRA sequences helped us to distinguish between the two. The discrimination of the major vessels of the head was also guided by [90]. Because of the detailed information on the vasculature, the use of the model may be advantageous in several applications, including thermal analysis [91]—where the impact of perfusion is high—and computational fluid dynamics.

The diffusion-weighted MRI acquisition was performed with an SSh-SE-EPI sequence with a high SENSE undersampling factor to achieve robustness against physiological motion while minimizing static field inhomogeneity distortions. The DTI measures diffusion as a 3D process and thereby reflects tissue characteristics such as the orientation of WM tracts. The inclusion of the local tissue anisotropy information that can be derived from the DTI data has been shown to be critical for accuracy in the forward/inverse problem related to electroencephalography (EEG) and magnetoencephalography (MEG) source localization [92] and in applications related to deep brain stimulation [93].

### Data Segmentation and Labeling

The second step of the image-based model generation procedure is the segmentation step, which consists of partitioning the images into non-overlapping constituent regions (*outlining*) and assigning unique labels to each of them according to anatomical atlases (*labeling*). Segmentation can be automatic, semi-automatic, or manual, and the accuracy of segmentation varies significantly as a function of the imaging modality, image quality, and the required accuracy of the results. A wide variety of automated approaches have been proposed for MRI segmentation [94]. Some anatomical structures contrast highly with respect to the surrounding tissues, e.g., ventricles vs. adjacent WM in T2, and can be segmented automatically. Conversely, the automatic algorithms fail to accurately segment more complex structures, because of a lack of clear edges, the presence of intensity inhomogeneity, and noise in the images [95]. Consequently, a trade-off between automation and knowledge-based interaction was required.

The proposed head model was generated by means of a semi-automatic segmentation approach, with subsequent manual refinement and adjustment based on anatomical atlases. For example, for the skull segmentation, the thicker cranial bones, i.e., frontal, occipital, temporal, and parietal bones, could be segmented with a semi-automatic, i.e. region growing, algorithm because of the sharp boundaries with the surrounding muscle and dura tissues. Conversely, many of the facial bones, such as the zygomatic bones, required extensive manual adjustment to remove gaps caused by the automatic segmentation tools that could not identify thin bone structures. Differentiating between anatomically correct cavities, e.g., the foramen spinosum and the jugular foramen, and erroneous gaps caused by automatic segmentation presented additional challenges. Therefore, manual slice-by-slice adjustments in each view were needed to successfully segment the facial bones and the base of the skull. Comparison of the 3D reconstruction of the model with atlas images of the skull during this process was critical for deciding whether to include or rather close a cavity in the structure.

**Fig 19:**
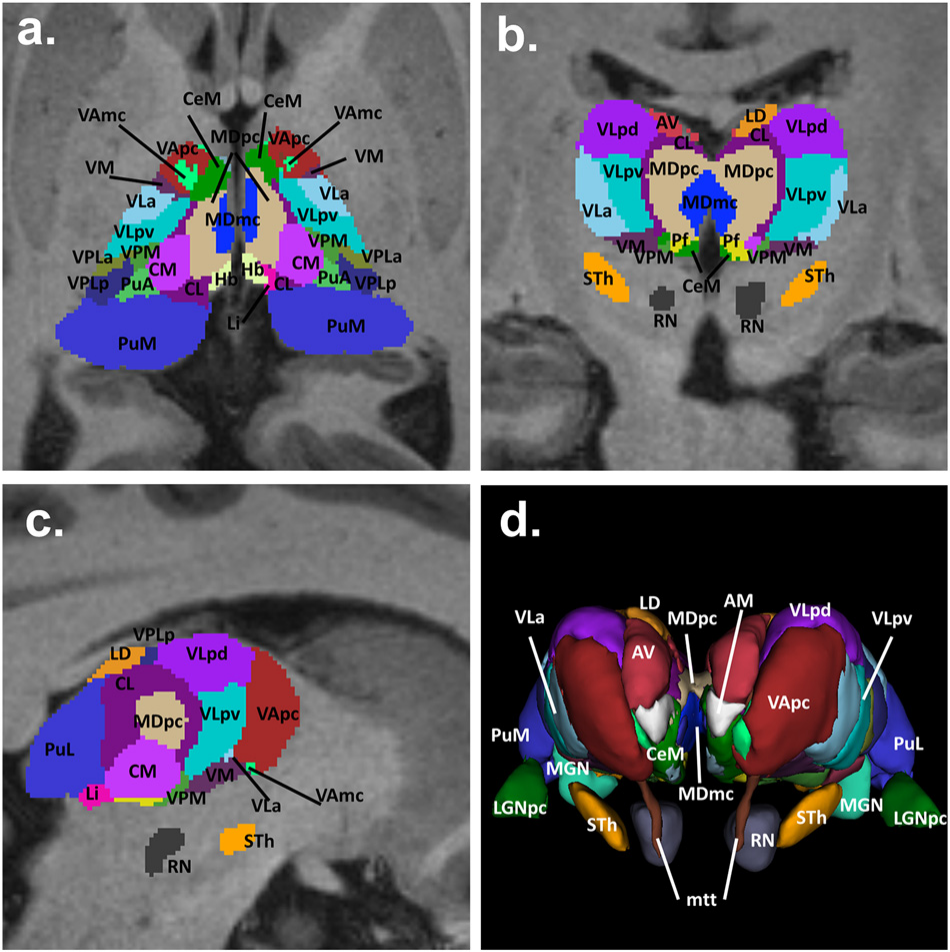
Segmentation of the thalamus and nuclei. Axial (a), coronal (b), sagittal (c), and 3D (d) views of the thalamus and nuclei. An automated atlas-based segmentation procedure was adopted to generate the map of the nuclei from the multiarchitectonic stereotactical atlas of the thalamus and to project them onto the head model.

Various atlases of the human brain have been reported in the literature for localization and segmentation of deep brain structures [96–100]. These atlases can be co-registered onto patient image data and be used to incorporate the contours of anatomical structures otherwise not visible on the images. Herein an automatic atlas-based segmentation was dedicated to the segmentation of the thalamus and 38 of its nuclei. Ground truth for the anatomy of thalamic nuclei was provided by the 3D statistical shape model of the thalamus by Anne Morel. Table 2 provides a list of the nuclei included in the segmentation. Fig 19 shows axial (a), coronal (b), and sagittal (c) slices of the segmentation of the nuclei overlaid onto the T1-weighted MRI image dataset. The 3D reconstructed model of the nuclei is also shown (d). The increased detail achieved in the region of the basal ganglia and the thalamus represents a potential key strength of the model for brain stimulation applications, in which the identification of the deep brain target nuclei with a high level of resolution is desirable [88,101].

### Surface Extraction

The methodology to extract and process surface meshes from the voxel data was based on specially developed algorithms, which produced high-quality triangle elements and topologically conformal surfaces. The latter aspect offers the advantage that gaps and overlaps between solids are avoided, and the creation of high-quality tetrahedral volume meshes, e.g., for FEM, is simplified. Indeed, intersections and self-intersections of the surfaces could invalidate the models in the meshing or voxeling discretization steps. The extensive manual refinement that accompanied the semiautomatic segmentation, together with the cleanup routines and the surface processing steps, allowed segmentation artifacts, such as staircasing, holes, and noise to be fixed and resulted in smoothed surfaces that maintain their anatomical fidelity and are optimal for unstructured tetrahedral mesh generation. Fig 6 shows the 3D surface reconstruction of representative structures of the head.

### Limitations

The determination of absolute anatomical accuracy and precision in the outlining of the anatomical structures has some limitations due to the lack of a segmentation ground truth. Segmentation errors may be due to the i) inadequacy of the images for the visualization of specific details, e.g., the lack of a specific MRI sequence for the deep brain structures, compensated partly by the integration of the Morel atlas, or inadequate spatial resolution for retina/choroid/sclera and vasculature imaging, e.g. minimal vessels diameters; ii) the presence of artifacts in the original data; iii) inaccuracies in the registration process used to estimate the alignment between MRA, eye/ear slab, and the non-isotropic DTI images, and in the atlas-based segmentation of the thalamus; iv) and discrepancies in the definitions of anatomical structures in the available literature, e.g., about brainstem division and the outlining of pons/cerebellum boundary; v) additional limitations include the use of a finite number of discrete tissues, while, in reality, tissue show continuous variation and inhomogeneity. Nevertheless, our chosen approach integrates both automatic and knowledge-based segmentation. The former method helps reduce the segmentation time and improve the quality of the results in terms of consistency, objectivity and, reproducibility, while the latter method minimizes the errors resulting from automatic classification by including expert knowledge about the anatomy. Inter-operator variability was assessed and showed to be non-significant and the results of the segmentation were reviewed by an expert anatomist.

Furthermore, our proposed model is subject-specific and does not take into account inter-individual anatomical variability. Currently, only a single head model has been generated. To capture inter-person variations, e.g., for population studies or to assess uncertainty related to anatomy, a series of independent models [50] spanning the range of the population of interest is needed and should be generated in the future. Alternatively, morphing procedures can be used to morph a previously segmented model to subject-specific image data. Nonetheless, even a single head model with such a detailed representation of the anatomy is valuable to formulate hypotheses, e.g., on interaction mechanisms, analyze the impact of parameters, or gain additional understanding on physical and physiological processes.

## Conclusions

We have developed MIDA, a comprehensive multi-modal model of anatomical structures of the human head and neck, distinguished by segmenting data integrated from three different MRI modality classes, namely MRI, MRA, and DTI. The underlying image data is characterized by a high resolution, up to 500 μm. Novel surface extraction and processing algorithms resulted in high quality, topologically conforming and non-(self) intersecting surfaces that facilitate the generation of volume meshes for FEM modeling. The unique multimodal high-resolution approach allowed 153 structures, including several distinct muscles, bones and skull layers, arteries and veins, as well as salivary glands, to be distinguished. The model offers also a detailed characterization of eyes, ears, deep brain structures, and an atlas-based segmentation of the nuclei of the thalamus and midbrain. The high resolution and the number of structures resolved in the model place MIDA among the most detailed state of the art image-based anatomical models. The model suitability to simulations involving different numerical methods and discretization approaches, as well as the impact of DTI-based tensorial electrical conductivity information on electromagnetic analysis, was ascertained in a specific application example, namely tACS. The voxel- and the surface-based versions of the model are freely available online at the following website: http://www.itis.ethz.ch/MIDA/ (DOI: 10.13099/ViP-MIDA-V1.0). Inquiries about the model can be sent to the following email address: MIDAmodel@fda.hhs.gov.

## Supporting Information

S1 Appendix S1 Appendix contains additional information on the semi-automatic segmentation methodology used for the model generation. Furthermore, specific information describing the methodology used to segment the following structures is included:
Skin: epidermis, dermis and subcutaneous tissue; Adipose tissue; Muscles; Skull, mandible, teeth, vertebrae, and intervertebral disks; The nasal structures and the internal air; Dura Mater; Cerebrum: gray matter, white matter, and CSF; Brainstem, Spinal Cord, and Cerebellum; Ventricular System; Deep brain structures; Nerves; Eye; Ear; Vessels; Salivary Glands.
(DOCX)
