# Supplementary material for: MIDA: A Multimodal Imaging-Based Detailed Anatomical Model of the Human Head and Neck
Source: PLoS One. 2015 Apr 22;10(4):e0124126. doi: 10.1371/journal.pone.0124126 (PMC4406723; doi:10.1371/journal.pone.0124126)
Supplement: S1 Appendix — Furthermore, specific information describing the methodology used to segment the following structures is included: Skin: epidermis, dermis and subcutaneous tissue; Adipose tissue; Muscles; Skull, mandible, teeth, vertebrae, and intervertebral disks; The nasal structures and the internal air; Dura Mater; Cerebrum: gray matter, white matter, and CSF; Brainstem, Spinal Cord, and Cerebellum; Ventricular System; Deep brain structures; Nerves; Eye; Ear; Vessels; Salivary Glands. (DOCX) [file pone.0124126.s001.docx]

# ‘Appendix

## iSeg

The iSeg software is a segmentation tool developed by the Foundation for Research on Information Technologies in Society software (Zurich MedTech, Zurich Switzerland) [[1](#_ENREF_1)]. The software implements a variety of operations to equip the user with semi-automatic and manual means of tissue discrimination. Segmentation operations performed on an original source image produce an output image that assigns a unique value to a discriminated region that can then be labeled as a particular tissue type. While iSeg provides additional segmentation tools, the following is a brief explanation of the tools and software features used to segment the tissues outlined in this report.

*Lock –* When assigning a new tissue type to a homogenous region in the target image, previously defined tissue types can be locked to prevent them from being overwritten as the new tissue.

*Thresholding –* A thresholding procedure attempts to determine an intensity value, called the threshold, which creates a binary partitioning of the image intensities. This technique is well suited when there are tissues with a clearly distinguished gray level range or when there is a clear anatomical distance to other tissues with similar gray values. There can be multiple thresholds to identify multiple regions (e.g., air, muscle, fat and bones) in one step. It is possible to specify the gray level boundary values manually or automatically by analyzing the histogram of the image and detecting `valleys' in the histogram which indicate that there are separable gray level classes.

*Region Growing –* This semi-automatic segmentation method creates a segmented region based on a seed placed on the source image by the user and intensity value limits. User-defined lower and upper gray value limits restrict the intensity values that are assigned to the segmented region. The segmented region includes pixels that fall within the limits that are connected to the seed point. The user also has the option to draw a limit onto the source image to restrict the area in which this operation is performed.

*Watershed –* This method requires the user to place one or more markers on the source image on the tissue one chooses to segment. Markers can even be placed on unconnected regions that can be defined as the same tissue. A slider is used to adjust the regions connected to the makers that will be defined as a tissue.

*K-means* – The k-means method requires a predefined number of tissue classes and use a minimization algorithm based on squared Euclidean distance to assign each voxel to a different class. Three parameters have to be provided when using these methods: the number of classes used for segmentation, the number of maximum iterations performed by the algorithm, and the convergence value which is used to determine when the algorithm has converged. This is considered to have occurred when less than the specified number of voxels has been reclassified in the last iteration.

*Morpho –* This function allows for the dilation, erosion or a combination of the two to expand or shrink the target image by a chosen number of pixels. The combinations include closing (expanding followed by shrinking), which closes small gaps and bays and opening (shrinking followed by expanding), which removes small islands and thin bridges between thicker regions. Morphological functions help expedite the smoothing process as they automatically create a more continuous structure.

*Interpol –* This tool allows for the interpolation between an initial and end slices. This function has the ability to automatically create segmented regions in the images between the first and last slice. For the purposes of this project, it is most effective to interpolate between two slices one tissue type at a time.

*Outline Correction (OLC): Brush –* The brush tool allows the user to manually make adjustments to the target or tissue image. The user is able to adjust the size of the brush radius and use it to add or remove pixels from the homogeneous regions in the target or tissue image.

*OLC: Fill All –* The fill option removes holes and gaps of any size within a homogeneous region.

*OLC: Add Skin –* This function adds an outline to a particular tissue type with a user-defined thickness in pixels or millimeters.

## Skin: epidermis, dermis and subcutaneous tissue

Human skin is composed of three distinct tissue layers: epidermis, dermis, and subcutaneous adipose tissue. While the epidermal thickness, 60 – 100 μm, is remarkably constant throughout the body, the dermis can range from 1 – 4 mm [[2](#_ENREF_2)]. The thickness values reported in the literature for the skin on the head range from 1.5 – 2 mm for the cheek and the forehead [[3](#_ENREF_3),[4](#_ENREF_4)], and are approximately 1.5 mm for other areas of the face, such as the auricular region, the scalp, and the neck. The skin around the eyes is less than 1 mm thick. The first step of the skin segmentation procedure was to remove the background noise and extract the mask of the head for successive partitioning and refinement. A median 3 × 3 × 3 voxel kernel filter was applied to the T1-weighted MRI image to remove the noise from homogenous areas while preserving the edges. A thresholding algorithm based on an intensity histogram analysis was then applied to extract the silhouette of the head from the background of the images. The result was finally smoothed according to the steps described in section 3 of the Materials and Methods.

In the proposed model, the epidermis and the dermis were not discernable and were segmented as a unique layer with an average constant thickness of 1.5 mm. The layer was obtained by thinning down the border of the mask of the head (“skin adding” option of iSeg). The deepest skin layer, namely the subcutaneous adipose tissue (SAT), is characterized by closely packed cells containing mostly fat. The boundary between this structure and the dermis is not well demarcated, and the thickness of the layer varies widely. Unlike the more superficial skin layers, the SAT appears as a bright layer, creating sufficient contrast with surrounding tissues for this structure to be segmented with the region growing algorithm. While this semi-automatic segmentation method allowed quick segmentation of the SAT in spite of the thickness variability, considerable manual refinements were necessary to create smooth contours. In addition, further refinements were necessary due to the presence of sparse muscle tissue in the SAT, particularly in the facial region, which made holes in the automatically segmented mask. To close the holes, some morphological operations, like closing, were used, followed by manual filling of the remaining gaps in the SAT mask.

## Adipose tissue

This tissue was initially segmented as the mask of the entire head; throughout the process of the segmentation, all other tissue types were discriminated, and the remainder was labeled as fat. These tissues consist mainly of adipose tissue – connective tissue cells that contain deposits of fat stored in their vesicles [[5](#_ENREF_5)].

## Muscles

The muscles found in these datasets pertain to the head, neck, and back. All muscles with the exception of the tongue muscles were initially painted as a single structure and then identified as individual muscles wherever feasible. Facial muscles – which are responsible for facial expressions – either originate or terminate in the dermal layers [[5](#_ENREF_5)], allowing the skin to be wrinkled and contracted. Furthermore, these muscles often merge at their sites of insertion and origination [[5](#_ENREF_5)], making their borders difficult to distinguish. As a result, segmentation of the facial muscles required extensive manual discrimination of individual muscles based on the T1-weighted dataset and anatomical atlases. Similar manual approaches were necessary for the neck muscles, which were characterized in the images by considerable noise and poor contrast due to motion during data acquisition. Conversely, the larger muscles, e.g., the posterior neck muscles, the dorsal muscles, and the muscles of mastication, which have clear borders and do not typically intersect the subcutaneous fasciae, could be segmented mostly automatically with moderate manual adjustment by means of the region growing algorithm. After manual refinement, larger muscles and muscles with clearly defined borders were distinguished and given individual tissue labels. Figure 14 shows the individually labeled muscles and the remaining muscles segmented as general muscles. A comprehensive list of the segmented muscles is provided in Table 1. Poor contrast between the muscles of the tongue and surrounding soft tissue made automatic segmentation of the tongue ineffective, such that manual segmentation was required.

## Skull, mandible, teeth, vertebrae, and intervertebral disks

The skull includes eight cranial bones – one frontal bone, one occipital bone, one sphenoid bone, one ethmoid bone, two temporal bones and two parietal bones – as well as six types of facial bones – two zygomatic bones, two nasal bones, two lacrimal bones, two palatine bones, one vomer and one maxilla. The cranial and some of the zygomatic and maxilla facial bones are shown in the 3D reconstruction of the bony structures in Figure 15 c and d. Each cranial bone contains three distinct layers: inner and outer tables, classified as cortical bone, and the cancellous diploë, which lies between the other two layers [[6](#_ENREF_6)]. The combined and individual layer thickness varies from bone to bone, with the thickest areas located in the occipital plate and the thinnest in the temporal plates; the thinnest diploë measurements also originate from the temporal plates [[7](#_ENREF_7)]. In MRI images, however, the diploë is the most easily differentiated layer of the three, due to its higher voxel intensity. Automatic segmentation algorithms were used to distinguish the thicker cranial bones from the surrounding muscle and dura tissue due to the high contrast between the dark tables and brighter surrounding tissues; these bones included the frontal, occipital, temporal, and parietal bones. Segmentation of thinner structures, such as the facial bones and the base of the skull, required considerable additional manual refinement.

Inferior to the maxillary bone of the skull, connected at the temporomandibular joint [[5](#_ENREF_5)], is the mandible, which is a cancellous bone. The mandible consists of two vertical rami that attach the curved horizontal body to the cranial bones. Sufficient contrast between the mandible and surrounding muscles allowed automatic segmentation methods to be used to distinguish the body and rami of the mandible, however manual refinement enabled additional discrimination of select details. Components that required manual segmentation include the mental and mandibular foramina – passageways for vessels and nerves through the mandible – and the roots of the teeth, which were distinguishable from the mandible only in the T2-weighted dataset. In addition, the manual smoothing techniques allowed the fibrous connective tissue to be distinguished from the bony tissues of the mandible and the coronoid process of the rami. To separate the two, manual adjustments according to [[8](#_ENREF_8),[9](#_ENREF_9)] were made.

The hyoid bone, like the mandible is an individual bone of the skull. Much like the mandible, the hyoid bone is a cancellous bone, but, due to size and close proximity to the poorly resolved neck area, this structure could not be segmented through automatic or semi-automatic methods. It was segmented manually according to [[8](#_ENREF_8),[10](#_ENREF_10)].

Embedded in the upper, i.e., maxillar, and lower, i.e., mandibular, jaws are 32 permanent teeth [[5](#_ENREF_5)]. Each tooth includes the three main components of crown, neck, and root [[6](#_ENREF_6)]. The root attaches the tooth to either the maxilla or mandible, leaving the crown to protrude out of the gingiva. In the T2-weighted dataset, the roots embedded in the maxillae and mandibles were visible, however the contrast between them was insufficient to allow automatic segmentation of the roots. The crowns and necks of individual teeth were segmented by means of an interactive watershed transformation algorithm, with markers placed on each tooth [[11](#_ENREF_11)]. The roots were distinguished from the jaws manually based on the visible contrast between the maxilla or mandible and the roots.

The final bony tissues included in the segmentation were the vertebrae, which provide support for the skull. The vertebrae visible in this dataset include the first five cervical vertebrae – C1 (atlas), C2 (axis), C3, C4, and C5. The initial process to segment these structures was performed in sagittal view, because the body of each vertebra was best discriminated in this view. The borders of these structures were not well enough defined to allow automatic segmentation, so these structures were differentiated manually on the basis of the visible contrast between the vertebrae and the surrounding tissues. Accepted definitions of vertebrae based on atlas data [[8-10](#_ENREF_8)] were also used to segment these structures. The atlases provide deeper insight regarding parts of the vertebrae that were discernible in neither the T1- nor the T2-weighted dataset, particularly the spinous processes and transverse foramina. Together with the vertebrae, the intervertebral disks, which are fibrocartilage structures situated contiguously between the vertebrae from the axis to the sacrum [[5](#_ENREF_5)], were segmented. As the primary connector of the vertebrae that allows movement of the vertebral column, the intervertebral discs are both strong and flexible. High contrast between the bony tissue of the vertebrae and the fibrocartilage allowed automatic and semi-automatic segmentation methods to be used.

## The nasal structures and the internal air

The complex anatomy of the nasal region includes several structures, including bone, mucosa, air, and cartilage (Figure 4). While the bony tissues were segmented as a part of the skull, the remaining tissues were segmented simultaneously by mean of the region growing algorithm in the T2-weighted dataset, where the nasal structures and internal air were more easily distinguishable. The mucosa envelops the bones to form the nasal cavity, which is lined with a moist tissue that filters, humidifies, and heats inhaled air to protect the lower respiratory tract [[12](#_ENREF_12)]. The nasal septum, which is comprised of bony and cartilaginous tissue and bisects the left and right nasal cavities, is the second structure that was simultaneously segmented in the T2-weighted dataset. While the bony components of the septum – the vomer and perpendicular plate of the ethmoid bone – were segmented as a part of the skull, the remaining cartilaginous tissue was extracted by means of the region growing algorithm and labeled as the nasal septum.

The skull contains several air-filled cavities. The internal air, contained in the nasal cavities, the frontal, maxillary, sphenoidal, and ethmoidal paranasal sinuses, the pharynx, and the mastoid sinus, was also segmented by means of the region growing algorithm in the T2-weighted dataset. With the exception of the mastoid sinus, all the sinuses converge in the nose; consequently, the internal air was segmented simultaneously with the nasal structures. The frontal sinus, maxillary sinuses, and anterior and middle ethmoid sinus air cells all drain into the nasal cavity through the middle meatus; posterior ethmoidal air cells drain through the superior meatus, and the spheno-ethmoid recess acts as the drainage route for the sphenoid sinus [[6](#_ENREF_6)]. There is much debate surrounding the nomenclature of the sinuses and their drainage systems [4, 11], however, in this model, the drainage routes are labeled as a part of the general internal air tissue.

Unlike the paranasal sinuses, the mastoidal sinus does not drain into the nasopharynx. Also known as the mastoid air cells, this sinus is a collection of air cavities of various sizes and shapes located in the mastoid process of the temporal bone [[5](#_ENREF_5)]. The mastoid air cell system works as a reservoir for the middle ear, exchanging gas through the mucosa that lines each individual cell [[13](#_ENREF_13),[14](#_ENREF_14)]. The individual cells differ morphologically: the superior cells tend to be greater in size and irregularity than the inferior cells [[5](#_ENREF_5)]. As the individual cells are not visible in any of the datasets, the mastoid air cell system was segmented as a single continuous structure.

## Dura Mater

The central nervous system is enveloped within three layers called meninges, which are, from the outermost layer inward, the dura mater, arachnoid, and pia mater. The dura mater, the thickest of the three layers, ranges between 0.2 – 0.5 mm thick depending on its cranial site [[15](#_ENREF_15)]. For this reason, the layer of the dura mater was the only one visible on the isotropic 500 µm MRIs. The dura mater was segmented by means of a region growing algorithm and was manually adjusted at some points where the layer appeared discontinuous due to the available 500 µm resolution.

Within the cranial vault, the dura mater presents also an inner meningeal surface that is reflected as sheet-like protrusions called dural septa [[16](#_ENREF_16)]. The falx cerebri, which partially separates the two cerebral hemispheres, and the tentorium cerebelli, which separates the superior surface of the cerebellum from the occipital lobes of the cerebrum, are the principal dural reflections (Figure 10). The falx was very fragmented and, thus, was only partially segmented. Conversely the tentorium was in large part visible and could be manually segmented. There are a few sites, where the outer and inner layers of the dura are not tightly fused and create internally intra-dural channels called dural venous sinuses, which empty into the cerebral veins [[9](#_ENREF_9),[16](#_ENREF_16)]. The dural sinuses were modeled by generating a 500 µm thick outer layer by the “skin adding” tool to envelop the large dural venous vessels visible on the MRA dataset, as explained in section 14 (Figure 10 and 18).

## Cerebrum: gray matter, white matter, and CSF

The cerebrum consists of two cerebral hemispheres, which make up the largest part of the brain. The cerebral cortex is a folded sheet of neurons and their interconnections that forms the corrugated surface of the cerebral hemispheres in a layer that varies from 1 – 4.5 mm thick and averages ca. 2.5 mm thick [[17](#_ENREF_17)]. The surface of the cortex is defined by GM convolutions, i.e., gyri, and depressions, i.e., sulci [[6](#_ENREF_6)] and overlies internal WM and the more deeply located basal ganglia. GM refers to areas where there is a preponderance of cell bodies and dendrites and WM to areas where there is a preponderance of myelinated axons composed mostly of lipids [[16](#_ENREF_16)]. In MRI, the presence of myelin causes a shortening of the longitudinal relaxation time (T1) that is more pronounced than the shortening of the transverse relaxation time (T2). The result of this phenomenon is that, compared to GM, WM appears hyperintense, i.e., with higher contrast, on T1-weighted images and isointense or slightly hypointense, i.e., with same or lower contrast, on T2-weighted images [[18](#_ENREF_18)]. The water content also influences the longitudinal relaxation time in the brain, with water-filled compartments characterized by longer T1, which means a lower intensity signal on T1-weighted MRIs. Figure 11 c shows a T1-weighted MRI of the brain where the low intensity signal for CSF appears dark, intermediate signal for GM appears gray, and the WM with the shortest T1 and strongest signal appears bright [[19](#_ENREF_19)].

Once the dura was outlined, it was used as the boundary edge to identify the space where the cerebrum lies. For this purpose, the outer dura was first used to isolate the intra-dural structures, i.e., the cerebrum, cerebellum, brainstem, and CSF, from the rest of the head. The intra-dural space was then converted into a binary mask (Figure 11 b) by assigning labels equal to zero and one to the voxels outside and inside the volume of interest. Multiplication of the original data with the binary mask data resulted in the generation of masked-MRIs that contain only the MRI intensities in the volume of interest (Figure 11 c). A *k*-means cluster analysis [[20](#_ENREF_20)] was then applied to classify the cerebrum tissues into three different classes: GM, WM, and CSF (Figure 11 d). The initial estimation of the centroids of the clusters was established by an automated histogram analysis procedure. Each voxel was then iteratively assigned to a specific tissue type by minimizing the within-cluster sum of squared Euclidean distances from the cluster centroid. The approach minimizes the overall within-cluster dispersion by iteratively reallocating cluster members until the convergence is smaller than a given threshold or after a maximum set number of iterations. The voxels of the cerebellar GM, which lie very close to the cortex GM and are of similar intensity, were misclassified as cerebrum GM by the automatic algorithm. The previous segmentation of the tentorium (Figure 10) was thus used as a boundary to distinguish the cerebellar structures from the cerebrum.

Unlike the layered structure of the cortex, the brainstem and the deep brain neuronal nuclei, which are dense nuclei of the GM, are connected by complex and intertwined axonal tracts below the resolution limits of standard MRI, which thus reduce contrast by partial volume averaging. These structures exhibit reduced contrast from WM in both T1- and T2-weighted images and are, thus, often misclassified by automatic segmentation algorithms [[21](#_ENREF_21),[22](#_ENREF_22)]. In this preliminary segmentation of the cerebrum, the brainstem and all the deep brain nuclei were included in the WM tissue type and were refined as individual structures in a successive step.

## Brainstem, Spinal Cord, and Cerebellum

The brainstem is the lower extension of the cerebrum, which forms the connection between it, the spinal cord, and cerebellum. The result of the *k*-means classification was used to further refine the brainstem and the spinal cord and to outline the structures of the brainstem, from the superior to the inferior, the midbrain, the pons, and the medulla oblongata, and the spinal cord. Figure 12 (right) shows the mid-coronal slice of the brainstem and the landmarks used for segmentation. The midbrain is characterized by four rounded prominences, i.e., the paired superior and inferior colliculi on its posterior surface, and by the cerebral peduncles on its anterior surface. The pons produces a prominent swelling with a well-defined interface with CSF on its anterior surface. The posterior surface of the pons is delimited by the floor of the fourth ventricle, which is bounded laterally by the middle cerebellar peduncle. Caudal to the pons is the medulla oblongata which is continuous caudally with the spinal cord.

The upper boundary of the brainstem, which corresponds to the upper boundary of the midbrain, was manually outlined as a surface passing through the posterior commissure posteriorly and the mammillary body anteriorly, with exclusion of the hypothalamic nuclei situated in the floor of the third ventricle [[23](#_ENREF_23)]. The remaining segmentation was performed by defining three cutting planes that represent the boundary between the structures shown by dashed blue lines in Figure 12 (right). The first plane, which represents the interface between the midbrain and the pons, was drawn on the mid-coronal plane so as to intersect the superior pontine notch and the inferior edge of the inferior colliculus. The second plane, i.e., the interface between the pons and the medulla, was drawn parallel to the first and intersecting the inferior pontine notch. The last plane to define the medulla oblongata and the spinal cord junction is one parallel to the previous planes at the level of the foramen magnum, which is an oval opening in the occipital bone of the skull where the vertebrae connect to the skull. Different integrative nuclei of the brainstem are present in the midbrain. Only the red nucleus was included in the model and was segmented together with the nuclei of the thalamus by means of an atlas-based segmentation as explained in section 4 of the Materials and Methods.

The tentorium layer and an imaginary plane that separates the segmented brainstem from the cerebellum were used to extract the subregion of cerebellar space, including the cerebellum and the CSF, from the intra-dural space. A *k*-means approach, like that proposed for the cerebrum, was applied on the cerebellar mask to classify the area into three classes: cerebellum GM, cerebellum WM, and CSF (Figure 12, middle). The three cerebellar peduncle pairs – superior, middle and inferior create a bridge of afferent and efferent fibers between the cerebellum and pons [[6](#_ENREF_6)]. As such, the peduncles were indistinguishable from the WM of the cerebellum, making atlas-based manual segmentation necessary [[24](#_ENREF_24)]. The peduncles were segmented as an extension of the cerebellum WM, rather than as individual structures.

## Ventricular System

The CSF fluid is a colorless liquid, low in cells and proteins, but otherwise generally similar to plasma in its composition. Figure 12 (left) shows the circulation of the CSF in the mid-coronal slice of the T1-weighted MRI dataset. Most of the CSF is produced within the ventricular system, primarily by the choroid plexus in the lateral ventricles, from where it passes through the interventricular foramina into the third ventricle, through the cerebral aqueduct into the fourth ventricle, and, thence, through the median and lateral apertures into the cisterna magna and the pontine cistern. The fluid slowly moves up over the cerebral hemispheres surrounding the brain into the subarachnoid space between the arachnoid mater and the pia mater. Finally, CSF is returned to the venous circulation through the superior sagittal sinus. In addition to this basic pattern of circulation, some CSF moves from the cisterns around the fourth ventricle into the subarachnoid space around the spinal cord. According to this view of CSF circulation, the volumes that are expected to contain CSF are the ventricular system (the two lateral ventricles, the third ventricle, the cerebral aqueduct, which is the connection between the third and fourth ventricle, and the fourth ventricle), the cisterns, the subarachnoid space over the brain, and the volume around the spinal cord.

The *k*-means approach was used to segment the CSF contained in the sulci and in the subarachnoid space, together with the cerebrum GM and WM, as described in Appendix S1, section 8. Neither refinement nor smoothing was applied because it was considered a fragmentary filling material.

The turbo spin echo (TSE) T2 dataset, because it yields high resolution and high signal-to-noise ratio (SNR) images with hyperintense CSF, was used for the segmentation of the ventricular system. For this reason, the ventricles and the cisterns were easily segmented by application of a region growing algorithm on the T2-weighted dataset. The segmentation of each slice was also checked for consistency with the T1-weighted dataset. The final result was smoothed by interpolation in between every three slices and application of morphological “closing” operations on the segmentation.

## Deep brain structures

The basal ganglia are a group of subcortical nuclei of GM deeply located in the basal region of the cerebral hemispheres. Anatomically and physiologically, the basal ganglia are connected to the thalamus and cerebral cortex through WM fiber pathways that convey motor, emotional, associative, and cognitive functions. The presence of WM tracts and the high iron content that characterize these nuclei are the cause of their reduced contrast in T1 and their slightly hypointense appearance with respect to the surrounding WM [[21](#_ENREF_21)]. In this study, the major nuclei of the basal ganglia, namely the caudate nucleus, the putamen, the nucleus accumbens and the globus pallidus, were segmented (Figure 13).

The segmentation of the caudate and the putamen was performed semi-automatically with a region growing algorithm and then refined on the coronal view of the MRIs according to standard atlases of the basal ganglia. The caudate nucleus is the largest nucleus of the basal ganglia and is characterized by a c-shaped structure composed of a large head, a narrowing body, and a curved thin tail. The head and the body of the caudate nucleus lie in between the lateral ventricles and the internal capsule bundle and were segmented on the basis of the intensity (Figure 13 a-h). The putamen and the pallidum were initially segmented as a single structure and were separated by manually outlining the thin layer of WM, known as external medullary lamina, between them (Figure 13 c-f). The nucleus accumbens was initially included in the caudate segmentation as it is not separable by visual inspection only. A border between the caudate and the accumbens nuclei was created by drawing a slightly oblique line from the inferior edge of the lateral ventricle laterally across the GM to the point where the internal capsule ends (Figure 13 b and c), as proposed in [[25](#_ENREF_25)]. The tiny tail of the caudate, located above the hippocampus, the caudo-putaminal bridge, and the internal medullary lamina – which separates the globus pallidus (GP) in its internal (GPi) and external (GPe) parts – were not discernable on the images and, therefore, were not included in the segmentation. Caudal to the basal ganglia are the hippocampus and the amygdala, which constitute the core of the limbic system. These structures were manually traced in coronal sequential slices based on the visual inspection and the use of anatomical atlases [[26](#_ENREF_26)]. The hippocampus is a bilaminar structure, shaped like a seahorse, formed by two intertwining folded layers of neurons, the cornu ammonis and the gyrus dentatus (Figure 13 g), and located symmetrically in the medial temporal lobes of the cerebrum. Macroscopically, the hippocampus can be divided into three segments along its longitudinal axis, the anterior part or head (Figure 13 f), the medial part or body (Figure 13 g and h), and the posterior part or tail (Figure 13 i). The landmarks used to trace the hippocampus are the alveus to delineate it anteriorly and superiorly, the trigone of the lateral ventricle posteriorly, the parahippocampal gyrus inferiorly, the inferior horn of the ventricle and the cisterna ambiens laterally and medially respectively (Figure 13 e-i). The amygdala was traced with the WM fiber bundles of the ansa peduncularis used as the superior border, the CSF and the entorhinal cortex as the medial border, and the WM as the lateral border; the inferior border was demarcated anteriorly by the parahippocampal gyrus and posteriorly by the alveus where the hippocampal head appears (Figure 13 d-f). The region growing algorithm was used to segment the hypothalamus, mamillary bodies, and the pituitary gland together on the axial view of the images. The pituitary gland appeared on the images as a hyperintense protrusion connected to the hypothalamus via a small tube called the infundibulum (Figure 13 c and d) [[27](#_ENREF_27)]. The borders of the pituitary gland were very sharp on the T1-weighted MRI, and the segmentation could be completed entirely automatically. The infundibulum was included in the segmentation as part of the pituitary. The mammillary bodies, which are adjacent to the hypothalamus (Figure 13 f), appeared only slightly hyperintense with respect to the surrounding tissues. However, their regular rounded shape allowed us to segment them easily by visual inspection. The segmented pituitary gland and mammillary bodies and the bright adjacent optic chiasm were used as external boundaries to outline the hypothalamus. Then, the hypothalamus was divided in the midline by the third ventricle. The segmentation of the subcortical structures also included the pineal gland, and the anterior and posterior commissures.

Finally, a specific automated atlas-based segmentation procedure to generate a map of the nuclei of the thalamus and midbrain from the multiarchitectonic stereotactic atlas of the thalamus [[28](#_ENREF_28)] and to project them onto the head model [[29](#_ENREF_29)] was adopted as explained in section 4 of the Materials and Methods (Figure 19).

## Nerves

There are 12 cranial nerves the nuclei of which originate in the brainstem [123]. As routine MRI is not optimal for nerve imaging [124], most of these nerves are poorly if at all visible in the available datasets. Visualization of the nerves is highly dependent on the thickness of the nerve itself; as such, various anatomical atlases were used to reasonably segment selected nerves when the MRI data was insufficient. In the cases of cranial nerves I, III, and VII, the olfactory, oculomotor, and facial nerves, respectively, only the nuclei were distinguishable, and we relied heavily on anatomical atlases for segmentation of the remaining portions of those nerves. The extratemporal portion of the facial nerve, cranial nerve VII, was not visible and not included in the segmentation.

Through the use of the ear-eye sequence slab and the T1-weighted datasets, the optic and vestibulocochlear nerves, cranial nerves II and VIII, respectively, could be segmented by routine automatic methods. Sufficient contrast provided by the ear-eye slab allowed these nerves to be clearly visualized.

Segmentation of the trigeminal nerve, cranial nerve V, required the use of atlases and additional manual segmentation to model the base of the nerve and its branches, the maxillary and mandibular divisions. The ophthalmic branch of the trigeminal nerve was not discernable on the images and was not included in the segmentation.

The intracranial portions of the abducens, glossopharyngeal, vagus, and hypoglossal nerves, cranial nerves VI, IX, X, and XII respectively, were visible using the ear-eye slab, while extracranial portions were segmented using anatomical atlases. The extracranial portion of the hypoglossal nerve, cranial nerve XII, was not discernable on the images and not included in the segmentation. The optic portion of the trochlear nerve, cranial nerve, IV, was visible using the ear-eye slab, while the remaining portion relied heavily on anatomical atlases. Segmentation of the accessory nerve, cranial nerve XI, also relied heavily on anatomical atlases. Figure 17 shows a 3D reconstruction of the cranial nerves included in the model.

## Eye

The eye is a round fluid-filled sensory organ, used to detect light and to image the visual scene, located inside the bony orbit of the skull. The globe of the eye is enveloped in a tough scleral tunic, which contains the aqueous and vitreous humors in the eye’s anterior and posterior chambers and helps maintain its rounded shape. The cornea and lens project images onto the thin sensory neural retina, which then sends visual signals through the optic nerve to the visual pathways of the brain. A series of six extraocular muscles attached to the eye operate in pairs in the periorbital fat – the superior/inferior rectus, lateral/medial rectus, and superior/inferior oblique – to direct the eye’s angle of gaze. The sixth extraocular muscle—the inferior oblique muscle – was segmented in the T1-weighted dataset manually based on [[6](#_ENREF_6)]. Manual refinements were made to smooth the remaining muscles and connect them to the sclera. The silhouette of the eye was easily extracted by application of a thresholding algorithm to the special ear/eye slab MRI. The substructures of the eye were then segmented by region growing from seed points placed in specific locations. The segmentation included the lens, the aqueous and vitreous humors, the cornea, the retina/choroid/sclera complex, the optic nerve, the chiasm, and the extraocular muscles (Figure 16, left). The interposed lens was the structure used to separate the two eye chambers. The retina, the choroid, and the sclera could not be resolved in the MRI scan, as no special ocular detector coils and eye motion reduction techniques [[30](#_ENREF_30)] were used, and thus, these structures were segmented as a single retina/choroid/scleral layer complex to form the posterior eye wall.

## Ear

The segmentation of the components of the ear – the pinna or auricula, cochlea, semicircular canals and vestibulocochlear nerve – included in this model was made possible primarily by the use of the ear/eye slab and the T2-weighted dataset (Figure 16 right). The pinna, the protruding portion of the external ear responsible for catching sound [[6](#_ENREF_6)], is composed of yellow elastic cartilage covered by skin [[5](#_ENREF_5)]. As the ear-eye slab does not encompass the cartilage of the pinna in its entirety, this tissue was segmented in the T2-weighted dataset. Semi-automatic, i.e., region growing, segmentation methods were of limited value in discerning the auricular cartilage due to the abundance of depressions and protrusions characteristic of this structure. Nonetheless, manual segmentation on a slice-by-slice basis was performed to smooth the contours of the auricular cartilage. The eye/ear slab was essential for the segmentation of the cochlea and the semicircular canal structures of the inner ear, which lead to the vestibulocochlear nerve. Both the cochlea and the semicircular canals, components of the osseous labyrinth, are hollow bony tissues coated with periosteum that were clearly visible in the eye/ear slab as bright structures. Use of the region growing algorithm enabled the initial segmentation of these structures as a single unit; the cochlea and semicircular canals meet at a central vestibule and cannot be automatically segmented as individual structures. Manual segmentation and smoothing were required to separate these two structures. Segmentation of the external auditory canal and the auditory tube (also named the external auditory meatus and the pharyngotympanic/Eustachian tube respectively) was completed manually based on [[10](#_ENREF_10)]. The pharyngotympanic tube acts as a channel of air between the pharynx and middle ear composed of both bony and cartilaginous tissue [[6](#_ENREF_6)]. While the external portion of the auditory canal is clearly visible as an air-filled space protruding into the ear, the width of the inner portion – the pharyngotympanic tube is relatively smaller making manual atlas-based segmentation necessary.

## Vessels

The TOF and PCA MRA sequences were optimized to distinguish between the arteries and the veins in the head. The TOF sequence encodes flow direction and was optimized to highlight blood flowing in cranial direction, which resulted mostly in arteries being visible. The PCA imaging was instead optimized to highlight vessels whose flow speed was in a specific velocity window. The velocity window was chosen to enhance imaging of slower flow vessels such as the veins and suppress the signal from the major fast flowing arteries. The arteries and veins were segmented using a region growing algorithm with the seeds points placed in multiple branches of the vasculature tree (Figure 18). However, the two sequences presented some overlapping information due to the existence of slow blood flow arteries and arteries whose blood flowed in a caudal direction. For this reason, the segmentation was limited to the largest vessels recognizable on the anatomical atlases that could be assigned to the artery or the vein classes.

## Salivary Glands

The salivary glands are three pairs of organs whose secretions aid in the digestion of food in the mouth [[5](#_ENREF_5)]. The three gland pairs – the parotid, submandibular and sublingual – were most discernable in the T1-weighted dataset where they were initially segmented using the region growing algorithm. Manual refinements were made to separate the glands from surrounding soft tissue (muscle and fat).

**References**

1. Neufeld E High resolution hyperthermia treatment planning PhD Thesis ETH No. 17947: Swiss Federal Institute of Technology, Zurich 2008.

2. Rushmer RF, Buettner KJ, Short JM, Odland GF The skin. Science. 1966; 154: 343-348.

3. Seidenari S, Pagnoni A, di Nardo A, Giannetti A Echographic evaluation with image analysis of normal skin: variations according to age and sex. Skin Pharmacol. 1994; 7: 201-209.

4. Hoffmann K, Stuücker M, Dirschka T, Goörtz S, El‐Gammal S, Dirting K, et al. Twenty MHz B‐scan sonography for visualization and skin thickness measurement of human skin. J Eur Acad Dermatol Venereol. 1994; 3: 302-313.

5. Gray H Gray's Anatomy of the Human Body: Lea & Febiger; 1973.

6. Schuenke M, Schulte E, Schumacher U THIEME Atlas of Anatomy: Head and Neuroanatomy: Thieme; 2010.

7. Law S Thickness and resistivity variations over the upper surface of the human skull. Brain Topogr. 1993; 6: 99-109.

8. Neuroanatomy Modules: Head, Neck, Brain, Spine; <http://headneckbrainspine.com/>

9. Netter F Atlas of Human Anatomy; III, editor; 2003.

10. e-Anatomy IMAIOS atlas; <http://www.imaios.com/>

11. Beucher S, Lantuejoul C. Use of watersheds in contour detection. In International Workshop on Image Processing: Real-time Edge and Motion Detection/Estimation. 1979.

12. Watelet JB, Cauwenberge PV Applied anatomy and physiology of the nose and paranasal sinuses. Allergy. 1999; 54: 14-25.

13. Park MS, Yoo SH, Lee DH Measurement of surface area in human mastoid air cell system. J Laryngol Otol. 2000; 114: 93-96.

14. Koç A, Ekinci G, Bilgili A, Akpinar I, Yakut H, Han T Evaluation of the mastoid air cell system by high resolution computed tomography: three-dimensional multiplanar volume rendering technique. J Laryngol Otol. 2003; 117: 595.

15. Willatt DJ, Yung MW, Helliwell TR A correlation of the surgical anatomy of the dura to head and neck surgery. Arch Otorhinolaryngol. 1987; 243: 403-406.

16. Nolte G Human Brain: An Introduction to its Functional Anatomy Mosby; 2008.

17. Fischl B, Dale AM Measuring the thickness of the human cerebral cortex from magnetic resonance images. Proc Natl Acad Sci U S A. 2000; 97: 11050-11055.

18. Barkovich AJ Concepts of myelin and myelination in neuroradiology. Am J Neuroradiol. 2000; 21: 1099-1109.

19. Paus T, Collins D, Evans A, Leonard G, Pike B, Zijdenbos A Maturation of white matter in the human brain: a review of magnetic resonance studies. Brain Res Bull. 2001; 54: 255-266.

20. Coleman GB, Andrews HC Image segmentation by clustering. Proc IEEE. 1979; 67: 773-785.

21. Helms G, Draganski B, Frackowiak R, Ashburner J, Weiskopf N Improved segmentation of deep brain grey matter structures using magnetization transfer (MT) parameter maps. Neuroimage. 2009; 47: 194-198.

22. Haacke EM, Cheng NY, House MJ, Liu Q, Neelavalli J, Ogg RJ, et al. Imaging iron stores in the brain using magnetic resonance imaging. Magn Reson Imaging. 2005; 23: 1-25.

23. Tamraz JC, Comair YG, Tamraz J Atlas of regional anatomy of the brain using MRI: Springer Berlin; 2000.

24. Naidich T (2009) Duvernoy’s Atlas of the Human Brain Stem and Cerebellum. Am Soc Neuroradiology.

25. Makris N, Angelone L, Tulloch S, Sorg S, Kaiser J, Kennedy D, et al. MRI-based anatomical model of the human head for specific absorption rate mapping. Med Biol Eng Comput. 2008; 46: 1239-1251.

26. Duvernoy HM The human hippocampus: functional anatomy, vascularization and serial sections with MRI: Springer; 2005.

27. Daniel PM Anatomy of the hypothalamus and pituitary gland. J Clin Pathol Suppl (Assoc Clin Pathol). 1976; 7: 1-7.

28. Morel A Stereotactic atlas of the human thalamus and basal ganglia: CRC Press; 2007.

29. Jakab A, Blanc R, Berényi E, Székely G Generation of Individualized Thalamus Target Maps by Using Statistical Shape Models and Thalamocortical Tractography. AJNR Am J Neuroradiol. 2012; 33: 2110-2116.

30. Zhang Y, Nateras OS, Peng Q, Kuranov RV, Harrison JM, Milner TE, et al. Lamina-specific anatomic magnetic resonance imaging of the human retina. Invest Ophthalmol Vis Sci. 2011; 52: 7232-7237.
